# Spontaneous symbiotic reprogramming of plant roots triggered by receptor-like kinases

**DOI:** 10.7554/eLife.03891

**Published:** 2014-11-25

**Authors:** Martina Katharina Ried, Meritxell Antolín-Llovera, Martin Parniske

**Affiliations:** Faculty of Biology, Ludwig Maximilians University Munich, Munich, Germany; Boyce Thompson Institute for Plant Research, United States

**Keywords:** *Lotus japonicus*, plant root symbiosis, receptor-like kinase, signal transduction, plant development, gene regulation, Other

## Abstract

Symbiosis Receptor-like Kinase (SYMRK) is indispensable for the development of phosphate-acquiring arbuscular mycorrhiza (AM) as well as nitrogen-fixing root nodule symbiosis, but the mechanisms that discriminate between the two distinct symbiotic developmental fates have been enigmatic. In this study, we show that upon ectopic expression, the receptor-like kinase genes *Nod Factor Receptor 1 (NFR1)*, *NFR5,* and *SYMRK* initiate spontaneous nodule organogenesis and nodulation-related gene expression in the absence of rhizobia. Furthermore, overexpressed NFR1 or NFR5 associated with endogenous SYMRK in roots of the legume *Lotus japonicus*. Epistasis tests revealed that the dominant active *SYMRK* allele initiates signalling independently of either the *NFR1* or *NFR5* gene and upstream of a set of genes required for the generation or decoding of calcium-spiking in both symbioses. Only *SYMRK* but not *NFR* overexpression triggered the expression of AM-related genes, indicating that the receptors play a key role in the decision between AM- or root nodule symbiosis-development.

**DOI:** http://dx.doi.org/10.7554/eLife.03891.001

## eLife digest

Like all plants, crop plants need nutrients such as nitrogen and phosphate to grow. Often these essential elements are in short supply, and so millions of tons of fertiliser are applied to agricultural land each year to maintain crop yields. Another way for plants to gain access to scarce nutrients is to form symbiotic relationships with microorganisms that live in the soil. Plants pass on carbon-containing compounds—such as sugars—to the microbes and, in return, certain fungi provide minerals—such as phosphates—to the plants. Some plants called legumes (such as peas, beans, and clovers) can also form relationships with bacteria that convert nitrogen from the air into ammonia, which the plants then use to make molecules such as DNA and proteins.

To establish these symbiotic relationships with plants, nitrogen-fixing bacteria release chemical signals that are recognized via receptor proteins, called NFR1 and NFR5, found on the surface of the plant root cells. These signals trigger a cascade of events that ultimately lead to the plant forming an organ called ‘root nodule’ to house and nourish the nitrogen-fixing bacteria. A similar signalling mechanism is thought to take place during the establishment of symbiotic relationships between plants and certain soil fungi.

A plant protein called Symbiosis Receptor-like Kinase (or SYMRK for short) that is also located on the root cell surface is required for both bacteria–plant and fungi–plant associations to occur. However, the exact role of this protein in these processes was unclear. Ried et al. have now investigated this by taking advantage of a property of cell surface receptor proteins: if some of these proteins are made in excessive amounts they activate their signalling cascades even when the initial signal is not present.

Ried et al. engineered plants called *Lotus japonicus* to produce high levels of SYMRK, NFR1, or NFR5. Each of these changes was sufficient to trigger the plants to develop root nodules in the absence of microbes. Genes associated with the activation of the signalling cascade involved the formation of root nodules were also switched on when each of the three proteins was produced in large amounts. In contrast, only an excess of SYMRK could activate genes related to fungi–plant associations. Ried et al. also found that, while SYMRK can function in the absence of the NFRs, NFR1 and NFR5 need each other to function. These data suggest that the receptor proteins play a key role in the decision between the establishment of an association with a bacterium or a fungus.

As an excess of symbiotic receptors caused plants to form symbiotic structures, Ried et al. propose that this strategy could be used to persuade plants that usually do not form symbioses with nitrogen-fixing bacteria to do so. If this is possible, it might lead us to engineer crop plants to form symbiotic interactions with nitrogen-fixing bacteria; this would help increase crop yields and enable crops to be grown in nitrogen-poor environments without the addition of extra fertiliser.

**DOI:** http://dx.doi.org/10.7554/eLife.03891.002

## Introduction

Plants circumvent nutrient deficiencies by establishing mutualistic symbioses with arbuscular mycorrhiza (AM) fungi or nitrogen-fixing rhizobia and *Frankia* bacteria (Gutjahr and Parniske, 2013; Oldroyd, 2013). One of the first steps in the reciprocal recognition between rhizobia and the legume *Lotus japonicus* is the perception of bacterial lipo-chitooligosaccharides, so called nodulation factors, by the two lysin motif (LysM) receptor-like kinases (RLKs) Nod Factor Receptor 1 (NFR1) and NFR5 (Madsen et al., 2003; Radutoiu et al., 2003, 2007; Broghammer et al., 2012). Nodulation factor application induces two genetically separable calcium signatures in root hair cells; an early transient influx into the cytoplasm and within minutes calcium-spiking - periodic calcium oscillations in and around plant cell nuclei (Ehrhardt et al., 1996; Miwa et al., 2006; Oldroyd, 2013). (Lipo)-chitooligosaccharides have also been isolated from AM fungi (Maillet et al., 2011; Genre et al., 2013) and a NFR5-related LysM-RLK from *Parasponia* has been pinpointed as a likely candidate for their perception (Op Den Camp et al., 2011). The common symbiosis genes of legumes are required for AM and root nodule symbiosis. A subset of these genes is essential for either the generation or the decoding of calcium-spiking. In *L. japonicus*, the former group encodes the RLK Symbiosis Receptor-like Kinase (SYMRK; Stracke et al., 2002; Antolín-Llovera et al., 2014), two cation-permeable ion channels CASTOR and POLLUX (Imaizumi-Anraku et al., 2005; Charpentier et al., 2008; Venkateshwaran et al., 2012) as well as the nucleoporins NUP85, NUP133, and NENA (Kanamori et al., 2006; Saito et al., 2007; Groth et al., 2010). The latter group encodes Calcium Calmodulin-dependent Protein Kinase (CCaMK; Tirichine et al., 2006; Miller et al., 2013) and CYCLOPS (Yano et al., 2008), which form a complex that has been implicated in the deciphering of calcium-spiking (Kosuta et al., 2008). Phosphorylation by CCaMK activates CYCLOPS, a DNA-binding transcriptional activator of the *NODULE INCEPTION* gene (*NIN*; Schauser et al., 1999; Singh et al., 2014). NIN itself is a legume-specific and root nodule symbiosis-related transcription factor and regulates the *Nuclear Factor-Y subunit* genes *NF-YA1* and *NF-YB1* that control the cell division cycle (Soyano et al., 2013; Yoro et al., 2014). The paradigm of a common signalling pathway for both symbioses bears important open questions about the molecular mechanisms that ensure the appropriate cellular response for AM fungi on the one hand and for rhizobia on the other hand.

SYMRK carries an ectodomain composed of a malectin-like domain (MLD), and a leucine-rich repeat (LRR) region that experienced structural diversification during evolution (Markmann et al., 2008) and is cleaved to release the MLD (Antolín-Llovera et al., 2014). Although SYMRK has been cloned several years ago (Stracke et al., 2002), its precise function in symbiosis is still enigmatic. While *nfr* mutants lack most cellular and physiological responses to rhizobia (Radutoiu et al., 2003), including nodulation factor-induced calcium influx and calcium-spiking, root hairs of *symrk* mutants respond with calcium influx to nodulation factor but not with calcium-spiking, and do not develop infection threads with rhizobia (Stracke et al., 2002; Miwa et al., 2006). Based on these phenotypic observations, *SYMRK* was positioned downstream of the *NFRs* (Radutoiu et al., 2003; Miwa et al., 2006). Importantly, it has not been conclusively resolved whether *SYMRK* plays an active signalling role in symbiosis or, alternatively, is involved in mechanical stress desensitisation (Esseling et al., 2004). To approach this issue, we built on the observation that specific mutations in, or over-abundance of mammalian receptor tyrosine kinases on the cell surface is linked with the development of some cancers caused by spontaneous receptor complex formation and inappropriate initiation of signalling (Schlessinger, 2002; Wei et al., 2005; Shan et al., 2012). We hypothesized that similar behaviour could be triggered by overexpression of symbiosis-related plant RLKs, providing a tool to further dissect the specific signalling pathways they address.

## Results

### Symbiotic *RLKs* trigger spontaneous formation of root nodules

**Figure 1.**
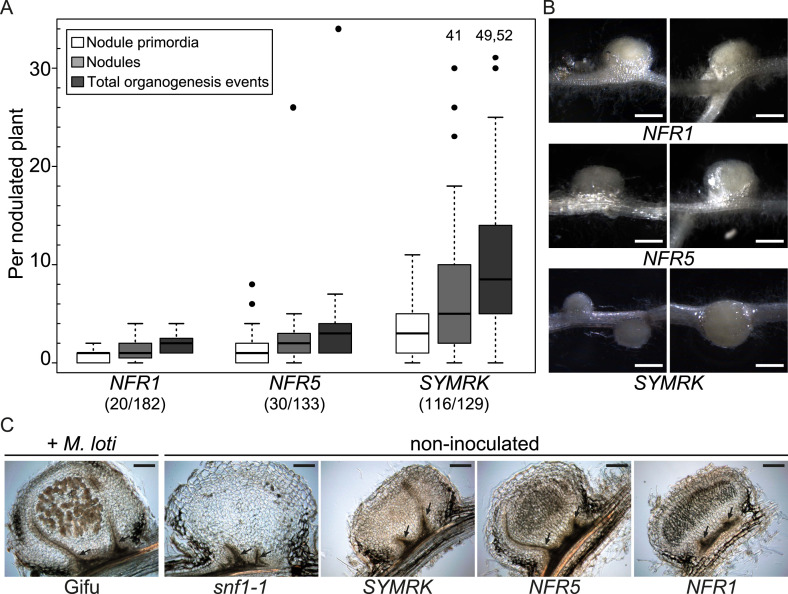
Symbiotic *RLKs* mediate spontaneous formation of root nodules. Hairy roots of *L. japonicus* Gifu wild-type transformed with the empty vector (EV), *pUB:NFR1-mOrange* (*NFR1*), *pUB:NFR5-mOrange* (*NFR5*), or *pUB:SYMRK-mOrange* (*SYMRK*) were generated. (**A**) Plot represents the numbers of nodule primordia (white), nodules (light grey) and total organogenesis events (dark grey; nodules and nodule primordia) per nodulated plant formed in the absence of rhizobia at 60 dpt. Number of nodulated plants per total plants is specified under each line label. Black dots, data points outside 1.5 interquartile range (IQR) of the upper quartile; numbers above upper whiskers indicate the values of individual data points outside of the plotting area. Bold black line, median; box, IQR; whiskers, lowest/highest data point within 1.5 IQR of the lower/upper quartile. Plants transformed with the empty vector did not develop spontaneous nodules. (**B**) Pictures of spontaneous nodules on hairy roots expressing the indicated transgenes taken 60 dpt. Bars, 1 mm. (**C**) Micrographs of sections of spontaneous nodules on hairy roots expressing the indicated transgenes harvested at 60 dpt. Spontaneous nodules of 10-week-old *snf1-1* mutant plants were used as controls. Nodules of 10-week-old untransformed *L. japonicus* wild-type Gifu 6 weeks after inoculation with *M. loti* MAFF303099 *Ds*RED contained cortical cells filled with bacteria (brown colour) that are absent in spontaneous nodules. Arrows point to peripheral vascular bundles. Longitudinal 40 mm sections. Bars, 150 µm. **DOI:** http://dx.doi.org/10.7554/eLife.03891.003

To achieve overexpression, we generated constructs expressing functional SYMRK (Antolín-Llovera et al., 2014), NFR5, or NFR1 under the control of the strong *L. japonicus Ubiquitin* promoter and added C-terminal mOrange fluorescent tags for detection purposes (*pUB:SYMRK-mOrange, pUB:NFR5-mOrange, pUB:NFR1-mOrange*). The functionality of the *NFR* constructs was confirmed by their ability to restore nodulation in the corresponding, otherwise nodulation deficient, *nfr* mutant roots to the level of *L. japonicus* wild-type roots transformed with the empty vector (Figure 1—figure supplement 1). Intriguingly, transgenic expression of any of the three symbiotic RLK versions in *L. japonicus* roots was sufficient to spontaneously activate the entire nodule organogenesis pathway as evidenced by the formation of nodule-like structures in the absence of rhizobia (Figure 1; Figure 1—figure supplement 2). The presence of peripheral vascular bundles instead of a central root vasculature unambiguously identified these lateral organs as spontaneous nodules (Figure 1C). Spontaneous nodule primordia or nodules were present on 90% (116 out of 129), 23% (30 out of 133), 11% (16 out of 182), and 0% (0 out of 164) of *L. japonicus* root systems at 60 days post transformation (dpt) with, respectively, *pUB:SYMRK-mOrange, pUB:NFR5-mOrange, pUB:NFR1-mOrange,* or the empty vector (Figure 1A; Figure 1—figure supplement 2). A total of 810 empty vector roots generated throughout the course of this study did not develop spontaneous nodules in any of the genetic backgrounds and time points tested. Roots expressing functional *SYMRK-RFP* from its native promoter (*pSYMRK:SYMRK-RFP*; Kosuta et al., 2011) and grown in the absence of rhizobia did not develop spontaneous nodules, indicating that spontaneous nodulation was triggered by *SYMRK* expression from the *Ubiquitin* promoter and not by the addition of a C-terminal tag alone (Figure 1—figure supplement 3). Moreover, the expression of non-tagged *SYMRK* under the control of the *Ubiquitin* promoter triggered the formation of spontaneous nodules. In comparison to roots transformed with the tagged *SYMRK* version, a lower number of roots transformed with non-tagged *SYMRK* contained spontaneous nodules (Figure 1—figure supplement 4). One explanation for this observation is that the C-terminal mOrange tag might result in alterations in the relative amount of signalling-active SYMRK. Another possibility is that the presence of the tag improves homo- and/or hetero-dimerization, which subsequently leads to downstream signalling. Our results demonstrate that overexpression of *NFR1-mOrange*, *NFR5-mOrange*, or *SYMRK* results in the activation and execution of the nodule organogenesis pathway in the absence of external symbiotic stimulation.

**Figure 1—figure supplement 1.**
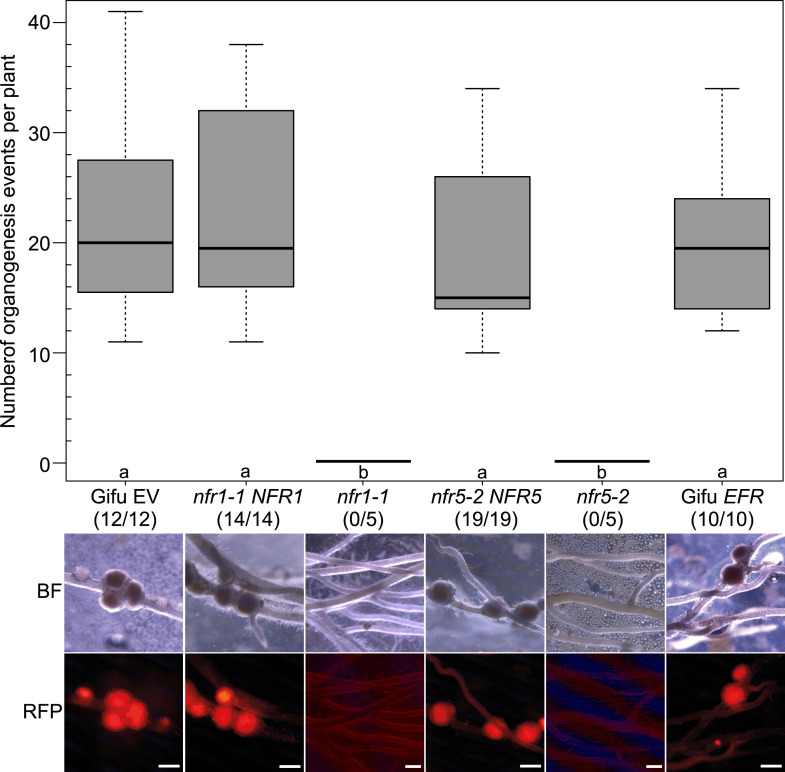
Expression of *NFR1* and *NFR5* from the *Ubiquitin* promoter restores nodulation in the *nfr1-1* and *nfr5-2* mutants, respectively. Hairy roots of *L. japonicus* Gifu wild-type transformed with the empty vector (EV) or with *pUB:EFR-mOrange* (*EFR*), the *nfr1-1* mutant transformed with *pUB:NFR1-mOrange* (*NFR1*) or the *nfr5-2* mutant transformed with *pUB:NFR5-mOrange* (*NFR5*) were generated. Untransformed *nfr1-1* and *nfr5-2* mutant plants served as control. Plot represents the number of organogenesis events (nodules and nodule primordia) per plant formed 15 days post inoculation with *M. loti Ds*RED. Numbers below each line label indicate the number of nodulated plants per total analysed plants. Representative pictures are shown. BF, bright field; RFP, RFP filter. Bars, 1 mm. Bold black line, median; box, IQR; whiskers, lowest/highest data point within 1.5 IQR of the lower/upper quartile. A Kruskal–Wallis test followed by false discovery rate correction was performed. Different letters indicate significant differences. p < 0.05. **DOI:** http://dx.doi.org/10.7554/eLife.03891.004

**Figure 1—figure supplement 2.**
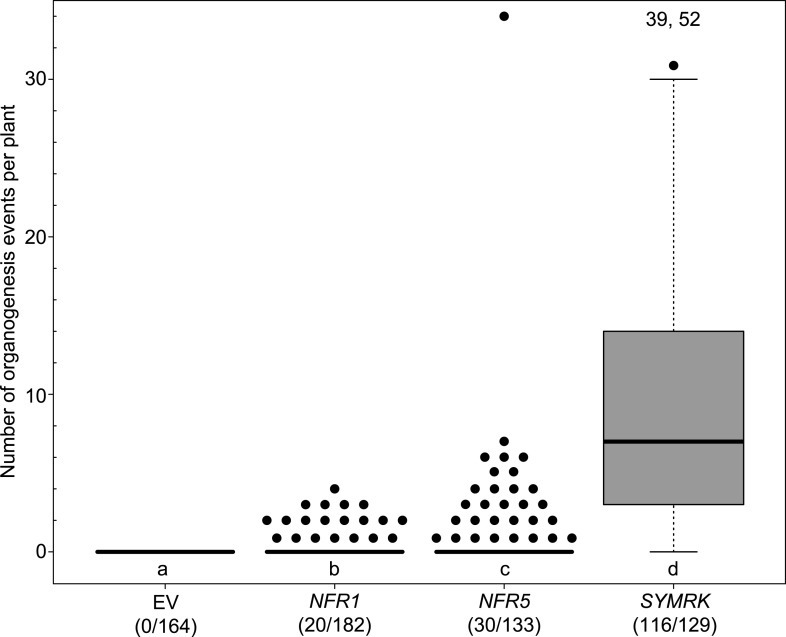
Statistical analysis of spontaneous root nodule formation. Hairy roots of *L japonicus* Gifu wild-type transformed with the empty vector (EV), *pUB:NFR1-mOrange* (*NFR1*), *pUB:NFR5-mOrange* (*NFR5*), or *pUB:SYMRK-mOrange* (*SYMRK*) were generated. Plot represents the numbers of organogenesis events (nodules and nodule primordia) per plant formed in the absence of rhizobia at 60 dpt. Number of nodulated plants per total plants is specified under each line label. Black dots, data points outside 1.5 IQR of the upper quartile; numbers above upper whiskers indicate the values of individual data points outside of the plotting area. Bold black line, median; box, IQR; whiskers, lowest/highest data point within 1.5 IQR of the lower/upper quartile. A Kruskal–Wallis test followed by false discovery rate correction was performed. Different letters indicate significant differences. p < 0.05. **DOI:** http://dx.doi.org/10.7554/eLife.03891.005

**Figure 1—figure supplement 3.**
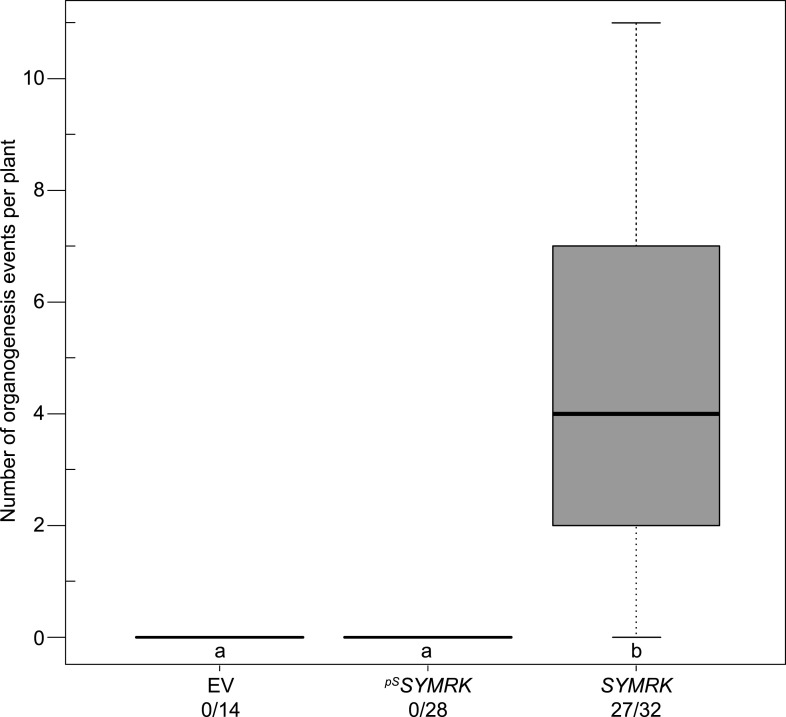
Expression of *SYMRK* from the native *SYMRK* promoter does not mediate spontaneous formation of root nodules. Hairy roots of *L. japonicus symrk-3* transformed with the empty vector (EV), *pUB:SYMRK-mOrange* (*SYMRK*), or *pSYMRK:SYMRK-RFP* (^*pS*^ *SYMRK*) were generated. Plot represents the number of total organogenesis events (nodules and nodule primordia) per plant formed in the absence of rhizobia at 21 dpt. Number of nodulated plants per total plants is specified under each line label. Bold black line, median; box, IQR; whiskers, lowest/highest data point within 1.5 IQR of the lower/upper quartile. A Kruskal–Wallis test followed by false discovery rate correction was performed. Different letters indicate significant differences. p < 0.05. **DOI:** http://dx.doi.org/10.7554/eLife.03891.006

**Figure 1—figure supplement 4.**
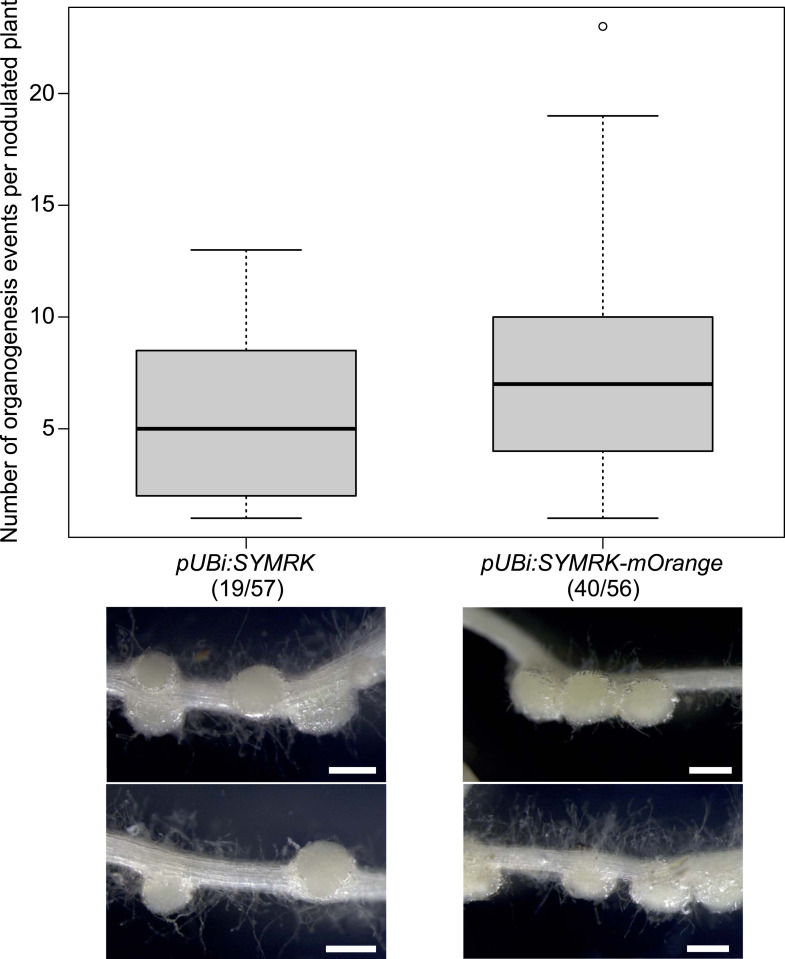
Expression of non-tagged *SYMRK* from the *Ubiquitin* promoter induces spontaneous formation of root nodules. Hairy roots of *L. japonicus symrk-3* transformed with *pUBi:SYMRK* (untagged) or *pUBi:SYMRK-mOrange* (C-terminally tagged) were generated. Plot represents the number of total organogenesis events (nodules and primordia) per nodulated plant formed in the absence of rhizobia at 42 dpt. Number of nodulated plants per total plants is specified under each line label. Dot, data point outside 1.5 interquartile range of the upper quartile. Bold black line, median; box, IQR; whiskers, lowest/highest data point within 1.5 IQR of the lower/upper quartile. Plants non-transformed or transformed with the empty vector did not develop spontaneous nodules. A Kruskal–Wallis test followed by false discovery rate correction was performed for total organogenesis events per nodulated root system (p-value of 0.16) and for total organogenesis events per transformed root system (p-value of 1.2e-05). Numbers below each line label indicate the number of nodulated plants per total analysed plants. Representative pictures are shown. Bars, 0.5 mm. **DOI:** http://dx.doi.org/10.7554/eLife.03891.007

### Symbiotic *RLKs* trigger spontaneous nodulation-related signal transduction

**Figure 2.**
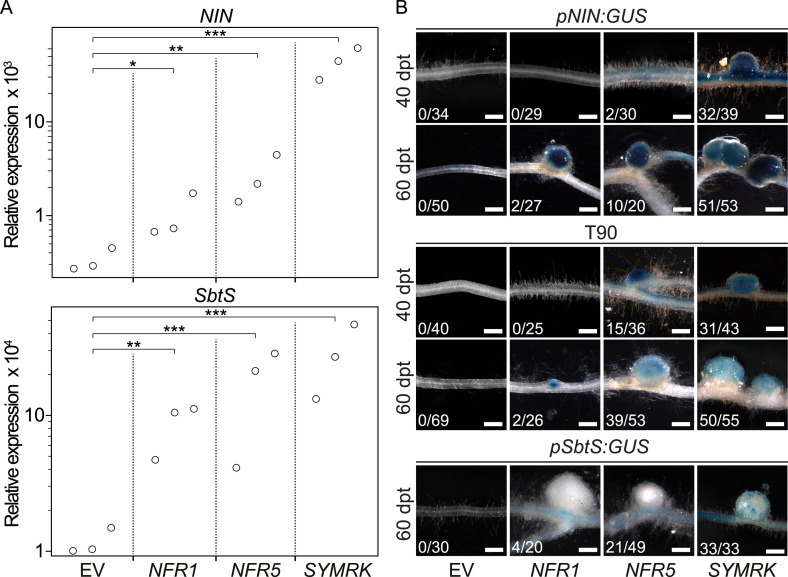
Symbiotic RLKs mediate spontaneous symbiosis-related signal transduction. Hairy roots of *L. japonicus* Gifu wild-type (**A**) or of three stable transgenic *L. japonicus* Gifu reporter lines (**B**)—carrying either the T90 reporter fusion, a *NIN* promoter:GUS fusion (*pNIN:GUS*), or a *SbtS* promoter:GUS fusion (*pSbtS:GUS*)—transformed with the empty vector (EV), *pUB:NFR1-mOrange* (*NFR1*), *pUB:NFR5-mOrange* (*NFR5*), or *pUB:SYMRK-mOrange* (*SYMRK*) were generated. (**A**) Relative expression of *NIN* or *SbtS* at 40 dpt was determined in three biological replicates for each treatment via qRT-PCR. Transcript levels in each replicate were determined through technical duplicates. Expression was normalized with the house keeping genes *EF1alpha* and *Ubiquitin*. Circles indicate expression relative to the *EF1alpha* gene. A Dunnett's test was performed comparing the transcript levels of *NIN* or *SbtS* detected for each treatment with those detected in the empty vector samples. Stars indicate significant differences from the EV control. *, p < 0.05; **, p < 0.01; ***, p < 0.001. (**B**) β-glucuronidase (GUS) activity was analysed by histochemical staining with 5‐bromo‐4‐chloro‐3‐indolyl glucuronide (X-Gluc) 40 and 60 dpt. Representative root sections are shown. Number of plants with detectable GUS activity per number of total plants is indicated. Bars, 500 μm. **DOI:** http://dx.doi.org/10.7554/eLife.03891.008

To establish whether the development of nodule-like structures was associated with nodulation-related gene activation, we analysed the expression behaviour of marker genes induced during root nodule symbiosis (*NIN* and *SbtS*; Kistner et al., 2005) via quantitative real-time PCR (qRT-PCR; Figure 2A). The *SbtS* gene is also induced during AM symbiosis (Kistner et al., 2005). In comparison to control roots transformed with the empty vector, the *SYMRK* construct resulted in a highly significant increase in *NIN* and *SbtS* transcript levels (mean fold increase of 137 and 24, respectively). A slighter but statistically significant increase in transcript levels could be observed in roots overexpressing either *NFR1-mOrange* (*NIN*, mean fold increase 3; *SbtS*, mean fold increase 7) or *NFR5-mOrange* (*NIN*, mean fold increase 8; *SbtS*, mean fold increase 15) (Figure 2A).

To monitor the spontaneous activation of *NIN* and *SbtS* by an independent and histochemical method, we made use of stable transgenic *L. japonicus* reporter lines carrying either a *NIN* promoter:*β-glucuronidase (GUS)* fusion (*pNIN:GUS;* Radutoiu et al., 2003) or a *SbtS* promoter:*GUS* fusion (*pSbtS:GUS*; Takeda et al., 2009) (Figure 2B). In addition, we employed the symbiosis-reporter line T90 that was isolated in a screen for symbiosis-specific *GUS* expression from a promoter-tagging population (Webb et al., 2000) (Figure 2B). The T90 reporter is activated in roots treated with nodulation factor or inoculated with *Mesorhizobium loti* and—similar to *pSbtS:GUS—*also shows *GUS* expression during AM (Radutoiu et al., 2003; Kistner et al., 2005). GUS activity was determined in roots by histochemical staining with 5-bromo-4-chloro-3-indolyl glucuronide (X-Gluc; Figure 2B). Either of the three symbiotic *RLKs* but not the empty vector activated the *pNIN:GUS,* the *pSbtS:GUS,* as well as the T90 reporter in the absence of *M. loti* or AM fungi (Figure 2B).

This histochemical analysis of GUS activity, in combination with the qRT-PCR results, provide strong evidence that overexpression of symbiotic *RLKs* leads to the activation of nodulation-related genes in the absence of external symbiotic stimulation (Figure 2). However, the three *RLK* genes were not equally effective in inducing the symbiotic program: *NFR5* or *NFR1* overexpression resulted in a lower percentage of root systems showing promoter activation and formation of spontaneous nodules when compared to *SYMRK* overexpression (Figure 1; Figure 1—figure supplement 2; Figure 2B). Interestingly, *SYMRK*- as well as *NFR5*-mediated T90 or *NIN* promoter activation was first observed in the root and retracted to nodule primordia and nodules over time, while *NFR1*-mediated T90 or *NIN* promoter activation could only be detected in nodule primordia or in nodules (Figure 2B). The *Ubiquitin* promoter drives expression of the receptors in all cells of the root (Maekawa et al., 2008), which is in marked contrast to the highly specific and developmentally controlled expression patterns of the marker genes observed. These incongruences thus reveal the presence of additional layers of regulation, operating downstream of the receptors, which dictate the precise expression patterns of the reporters.

### *SYMRK* triggers spontaneous AM-related signal transduction

**Figure 3.**
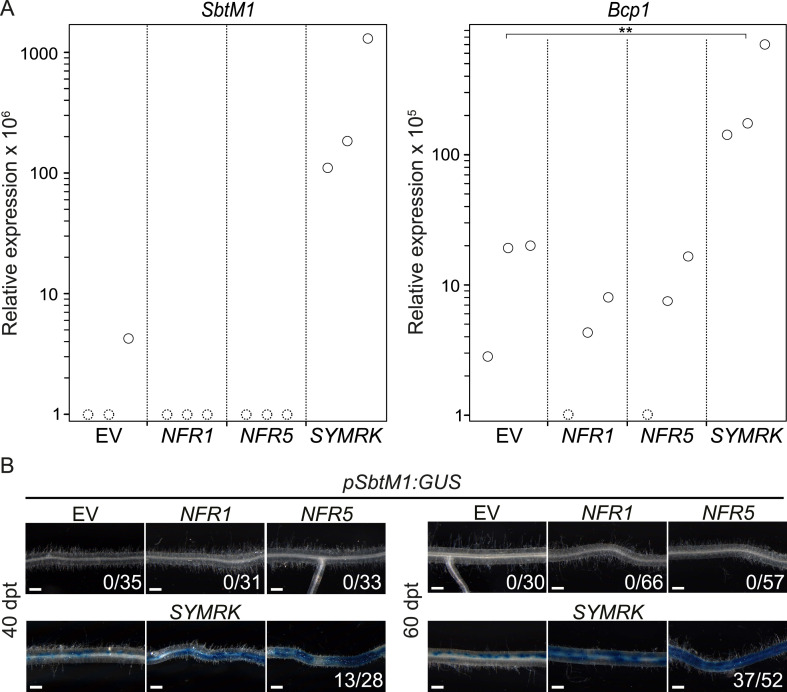
*SYMRK* mediates spontaneous AM-related signal transduction. Hairy roots of *L. japonicus* Gifu wild-type (**A**) or a stable transgenic L. *japonicus* MG20 reporter line carrying a *SbtM1* promoter:*GUS* fusion (*pSbtM1:GUS*) (**B**) transformed with the empty vector (EV), *pUB:NFR1-mOrange* (*NFR1*), *pUB:NFR5-mOrange* (*NFR5*), or *pUB:SYMRK-mOrange* (*SYMRK*) were generated. (**A**) Relative expression of *SbtM1* or *Bcp1* at 40 dpt was determined in three biological replicates for each treatment via qRT-PCR. Transcript levels in each replicate were determined through technical duplicates. Expression was normalized with the house keeping genes *EF1alpha* and *Ubiquitin*. Circles indicate expression relative to the *EF1alpha* gene. Dashed circles indicate that no transcripts could be detected for this sample. Samples in which the indicated transcript could not be detected were floored to 1. A Dunnett's test was performed comparing the transcript levels of *Bcp1* detected for each treatment with those detected in the empty vector samples. Stars indicate significant differences. **, p < 0.01. (**B**) GUS activity was analysed by histochemical staining with X-Gluc 40 and 60 dpt. Representative root sections are shown. Number of plants with detectable GUS activity per total plants is indicated. Bars, 500 μm. **DOI:** http://dx.doi.org/10.7554/eLife.03891.009

Since *SYMRK* is not only required for nodulation but also for AM symbiosis, we investigated the potential of dominant active *RLK* alleles to spontaneously activate AM-related marker genes or a promoter*:GUS* reporter (Figure 3). *Blue copper-binding protein 1* (*Bcp1*) and the subtilisin-like serine protease gene *SbtM1* are induced during AM symbiosis (Liu et al., 2003; Kistner et al., 2005; Takeda et al., 2009), and both genes are predominantly expressed in arbuscule-containing and adjacent cortical cells (Hohnjec et al., 2005; Takeda et al., 2009, 2012). Furthermore, in *L. japonicus*, *SbtM1* expression marks root cells that contain an AM fungi-induced prepenetration apparatus (Takeda et al., 2012)—an intracellular structure that forms prior to invasion by fungal hyphae (Genre et al., 2005). Transcript levels of *SbtM1* and *Bcp1* were determined via qRT-PCR, and both were significantly increased in roots transformed with *pUB:SYMRK-mOrange* compared to the empty vector control (Figure 3A). To determine *SbtM1* activation by an independent, histochemical approach, we employed a stable transgenic *L. japonicus* line harbouring a *SbtM1* promoter:*GUS* fusion (*pSbtM1:GUS;* Takeda et al., 2009). In line with the results from the qRT-PCR experiments, overexpression of *SYMRK-mOrange* in roots of the *pSbtM1:GUS* reporter line resulted in activation of the *SbtM1* promoter at 40 and 60 dpt (Figure 3B). In contrast, no *SbtM1* promoter activation or AM-related gene induction could be detected upon overexpression of either of the *NFRs* (Figure 3)*.* The absence of AM-related gene expression in *NFR5*-expressing roots is not a consequence of the overall lower induction power of the *NFR5* construct. In *SYMRK-* vs *NFR5*-expressing roots, the relative ratio of transcripts was 1.6:1 for *SbtS* and 17:1 for *NIN* (Figure 2A). In contrast, *SbtM1* was undetectable in *NFR5*- but more than 1100-fold above detection limit in *SYMRK-*overexpressing roots (Figure 3A). These data clearly demonstrate a strong difference in the gene repertoire activated by *SYMRK* vs *NFR5*. Together with the spontaneous nodulation, these results demonstrate that overexpression of *NFR1-mOrange, NFR5-mOrange,* or *SYMRK-mOrange* activates the nodulation pathway as evidenced by spontaneous organogenesis and gene expression results at the level of endogenous transcripts as well as promoter:*GUS* expression. In contrast, only the *SYMRK* construct but neither of the *NFR* constructs induced AM-related gene expression. This suggests that signalling specificity towards the two different symbiotic programs is achieved at the level of the receptors.

### SYMRK associates with NFR1 and NFR5 in *Lotus japonicus* roots

**Figure 4.**
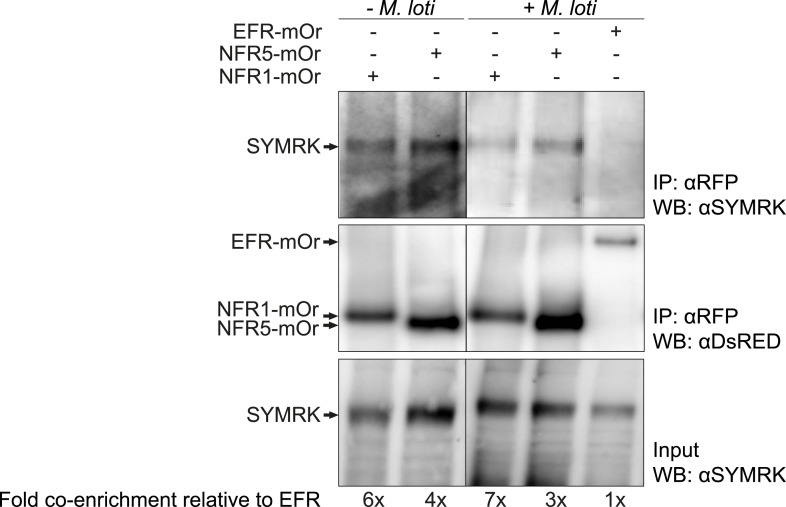
SYMRK associates with NFR1 and NFR5 in *Lotus japonicus* roots. Hairy roots of *L. japonicus* Gifu wild-type roots expressing *NFR1-mOrange* (NFR1-mOr), *NFR5-mOrange* (NFR5-mOr), or *EFR-mOrange* (EFR-mOr) under the control of the *Ubiquitin* promoter were extracted 10 days post inoculation with *M. loti Ds*RED or mock treatment. mOrange fusions were affinity bound with RFP magneto trap, and immuno-enrichment was monitored by immunoblot with and anti*Ds*RED antibody. Co-enrichment of endogenous SYMRK protein was monitored by immunoblot with an antiSYMRK antibody. Numbers below the western blot panels indicate the fold co-enrichment of SYMRK by NFR1 or NFR5 relative to the amount of SYMRK co-enriched with EFR. mOr, mOrange; IE, immuno-enrichment; WB, western blot. **DOI:** http://dx.doi.org/10.7554/eLife.03891.010

Spontaneous receptor complex formation caused by overexpression offers itself as a likely explanation for the observed activation of symbiosis signalling in the absence of an external trigger or ligand. This is a scenario described in the context of cancer formation, where receptor tyrosine kinase overexpression or specific mutations in the receptor lead to receptor dimerization in the absence of a ligand, which results in ectopic cell proliferation (Schlessinger, 2002; Wei et al., 2005; Shan et al., 2012). Upon expression in *Nicotiana benthamiana* leaves in the absence of symbiotic stimulation, we observed previously weak association between full-length SYMRK and NFR1 as well as NFR5, but not between SYMRK and the functionally unrelated RLK Brassinosteroid Insensitive 1 (BRI1; Li and Chory, 1997; Antolín-Llovera et al., 2014; Figure 4—figure supplement 1). To test whether overexpression is associated with receptor complex formation in *L. japonicus* roots, we employed the overexpression constructs of *NFR1*, *NFR5*, or the unrelated *EF-Tu receptor kinase* (*EFR*; Zipfel et al., 2006) for co-immuno-enrichment experiments. The *EFR* construct did not interfere with nodulation in wild-type plants (Figure 1—figure supplement 1). Endogenous full-length SYMRK was co-enriched with NFR1 and NFR5, but not with EFR demonstrating association of SYMRK and both NFRs (Figure 4). However, it should be noted that the expression strength of EFR was lower than that of NFR1 and NFR5. SYMRK-NFR association was detected in the absence of nodulation factor. We did not observe an effect of *M. loti* on this association at 10 days post inoculation (Figure 4).

**Figure 4—figure supplement 1.**
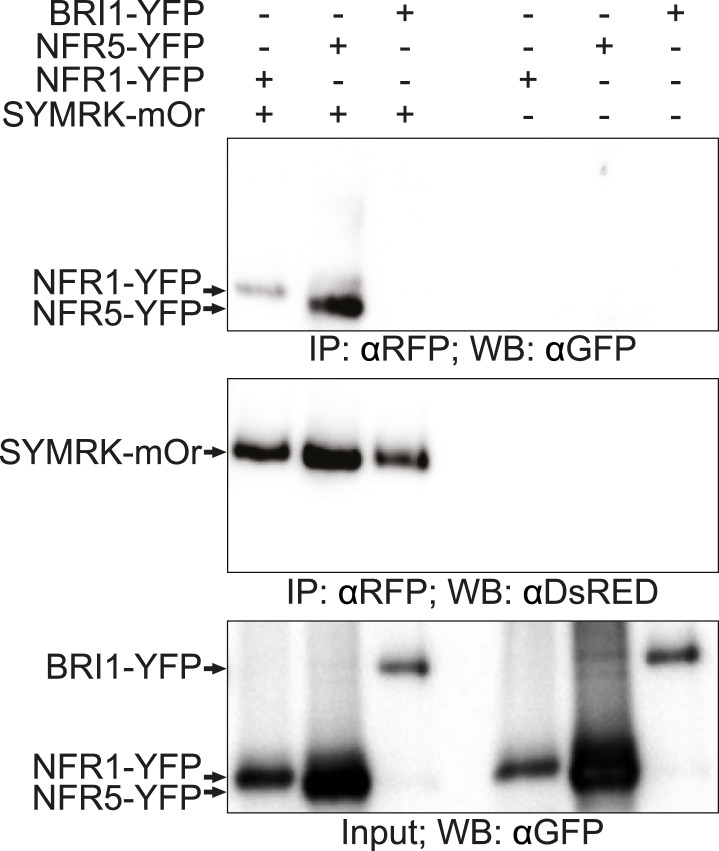
Full-length SYMRK associates with NFR1 and NFR5 in *Nicotiana benthamiana* leaves. *N. benthamiana* leaves were transiently co-transformed with constructs expressing NFR1-YFP, NFR5-YFP, or BRI1-YFP together with SYMRK-mOrange under the control of the CaMV 35S promoter. Leaf discs expressing the respective constructs were extracted 3 dpt. SYMRK-mOrange was immuno-enriched with RFP magnetotrap and monitored by immunoblot with an anti*Ds*RED antibody. Co-enrichment of NFR1-YFP, NFR5-YFP, or BRI1-YFP was monitored by immunoblot with an antiGFP antibody. mOr, mOrange; IE, immuno-enrichment; WB, western blot. **DOI:** http://dx.doi.org/10.7554/eLife.03891.011

### Epistatic relationships between *SYMRK* and other common symbiosis genes

**Figure 5.**
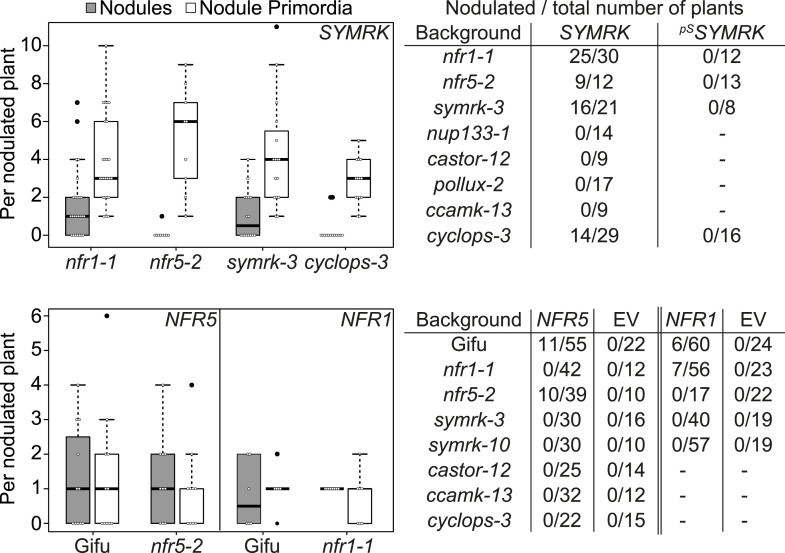
Epistatic relationships between symbiotic *RLK* genes and common symbiosis genes. Hairy roots of *L. japonicus* Gifu wild-type and different symbiosis defective mutants transformed with *pUB:SYMRK-mOrange* (*SYMRK*) or *pSYMRK:SYMRK-RFP* (^*pS*^ *SYMRK*) (upper panel), or the empty vector (EV), *pUB:NFR1-mOrange* (*NFR1*) or *pUB:NFR5-mOrange* (*NFR5*) (lower panel) were generated. Plots represent the numbers of nodules (grey) and nodule primordia (white) per nodulated plant formed in the absence of rhizobia at 40 (*SYMRK*) and 60 (*NFR5* + *NFR1*) dpt. White circles indicate individual organogenesis events. Black dots, data points outside 1.5 IQR of the upper/lower quartile; bold black line, median; box, IQR; whiskers, lowest/highest data point within 1.5 IQR of the lower/upper quartile. Table, fraction of nodulated per total number of plants. Plants transformed with *pSYMRK:SYMRK-RFP* or the empty *pUB* vector did not develop spontaneous nodules. **DOI:** http://dx.doi.org/10.7554/eLife.03891.012

The availability of dominant active receptor gene alleles offers an attractive tool for their positioning in the genetic pathway required for nodule organogenesis and symbiosis-related gene expression. We asked whether the *pUB:SYMRK-mOrange* construct induced spontaneous nodules or the symbiosis-specific T90 reporter in mutants of common symbiosis genes (Figure 5; Figure 5—figure supplement 1 and 4). *SYMRK*-induced spontaneous nodules were absent from *pollux-2, castor-12, nup133-1,* or *ccamk-13* mutant roots. Likewise T90 reporter (GUS) activation was not detectable in the *castor-2* x T90 (Kistner et al., 2005) or *ccamk-2* x T90 (Gossmann et al., 2012) lines (Figure 5—figure supplement 4). This epistasis revealed that the ion channel genes *CASTOR* and *POLLUX*, the nucleoporin gene *NUP133*, and the calcium- and calmodulin-dependent protein kinase gene *CCaMK*, operate downstream of *SYMRK* in a pathway leading to spontaneous nodulation and activation of T90 (Figure 5; Figure 5—figure supplement 1 and 4). In contrast, *SYMRK* induced spontaneous nodules on *cyclops-3* mutant roots (Figure 5; Figure 5—figure supplement 1). Spontaneous nodule formation on the *cyclops-3* mutant (Figure 5; Figure 5—figure supplement 1) corresponds to the formation of bump-like structures upon inoculation with *M. loti* on *cyclops* mutants (Yano et al., 2008). While bacterial infection is strongly impaired in *L. japonicus cyclops* or *M. truncatula ipd3* mutants, nodule primordia or nodules, respectively, develop upon rhizobia inoculation (Yano et al., 2008; Horvath et al., 2011; Ovchinnikova et al., 2011). Furthermore, an autoactive version of CCaMK is able to induce the formation of mature spontaneous nodules in *cyclops* mutants (Yano et al., 2008). The ability of *SYMRK* to mediate spontaneous nodule organogenesis in the *cyclops* mutant is consistent with these results and points towards the existence of redundancies in the genetic pathway leading to organogenesis at the level of *CYCLOPS* (Singh et al., 2014).

**Figure 5—figure supplement 1.**
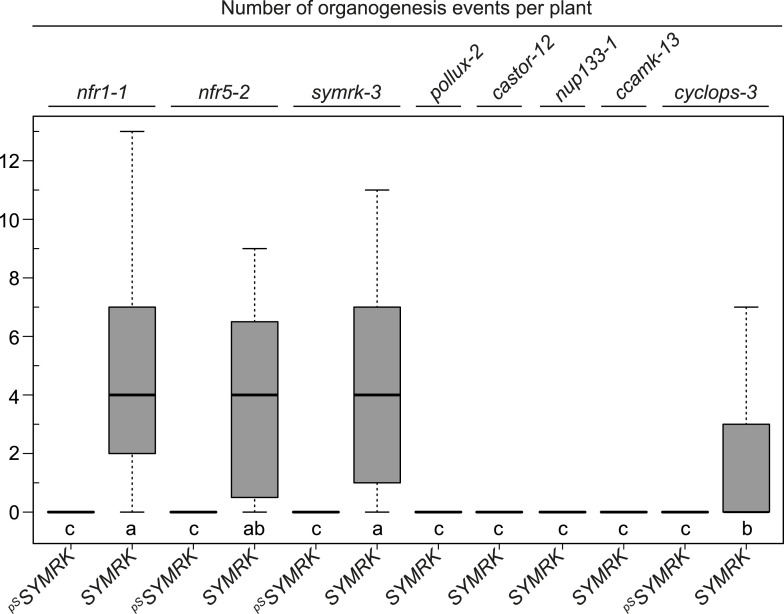
*SYMRK*-mediated spontaneous organogenesis events in *nfr1-1, nfr5-2*, and common symbiosis mutants. Hairy roots of different symbiosis defective mutants transformed with *pUB:SYMRK-mOrange* (*SYMRK*) or *pSYMRK:SYMRK-RFP* (^*pS*^ *SYMRK*) were generated. Plot represents the numbers of organogenesis events (nodules and nodule primordia) per plant formed in the absence of rhizobia at 40 dpt. Bold black line, median; box, IQR; whiskers, lowest/highest data point within 1.5 IQR of the lower/upper quartile. A Kruskal–Wallis test followed by false discovery rate correction was performed. Different letters indicate significant differences. p < 0.05. **DOI:** http://dx.doi.org/10.7554/eLife.03891.013

**Figure 5—figure supplement 2.**
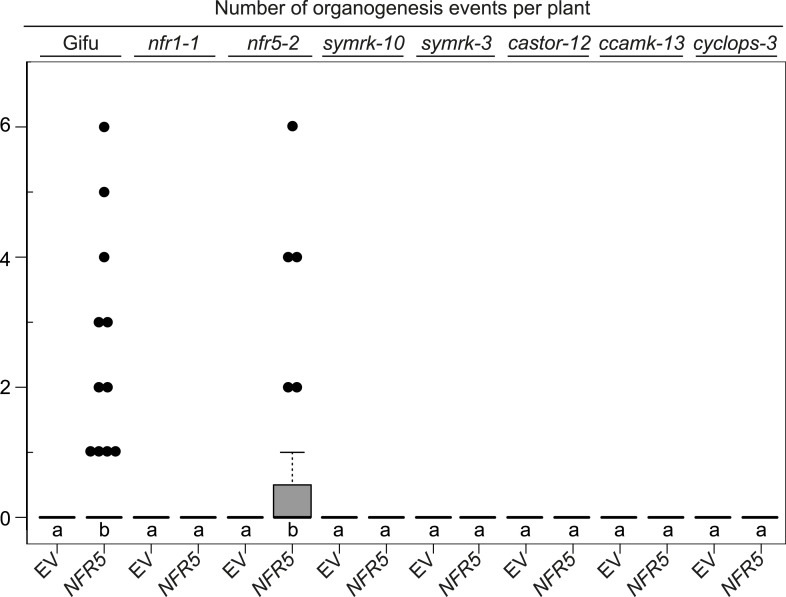
*NFR5*-mediated spontaneous organogenesis events in Gifu wild-type, *nfr1-1, nfr5-2*, and common symbiosis mutants. Hairy roots of *L. japonicus* Gifu wild-type and different symbiosis defective mutants transformed with the empty vector (EV) or *pUB:NFR5-mOrange* (*NFR5*) were generated. Plot represents the number of organogenesis events (nodules and nodule primordia) per plant formed in the absence of rhizobia at 60 dpt. Black dots, data points outside 1.5 IQR of the upper quartile; bold black line, median; box, IQR; whiskers, lowest/highest data point within 1.5 IQR of the lower/upper quartile. A Kruskal–Wallis test followed by false discovery rate correction was performed. Different letters indicate significant differences. p < 0.05. **DOI:** http://dx.doi.org/10.7554/eLife.03891.014

**Figure 5—figure supplement 3.**
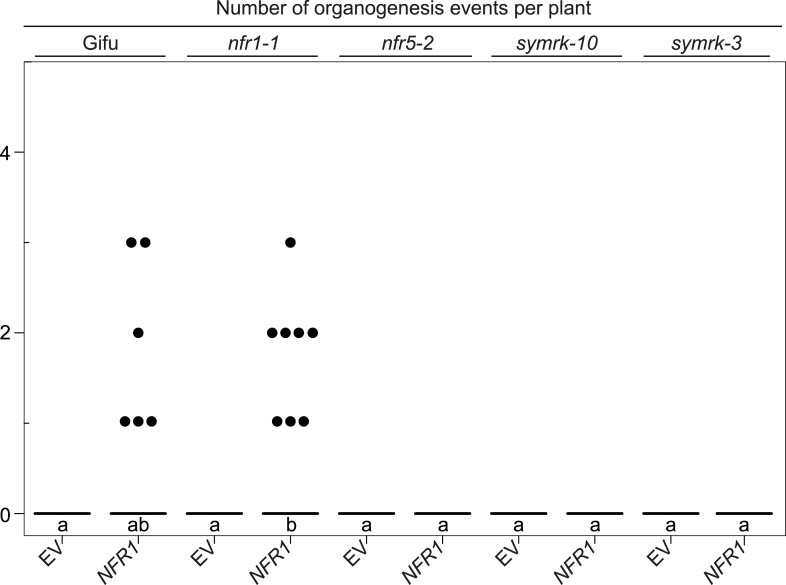
*NFR1*-mediated spontaneous organogenesis events in Gifu wild-type, *nfr1-1, nfr5-2*, *symrk-10,* and *symrk-3*. Hairy roots of *L. japonicus* Gifu wild-type and different symbiosis defective mutants transformed with the empty vector (EV) or *pUB:NFR1-mOrange* (*NFR1*) were generated. Plot represents the number of organogenesis events (nodules and nodule primordia) per plant formed in the absence of rhizobia at 60 dpt. Black dots, data points outside 1.5 IQR of the upper quartile; bold black line, median. A Kruskal–Wallis test followed by false discovery rate correction was performed. Different letters indicate significant differences. p < 0.05. **DOI:** http://dx.doi.org/10.7554/eLife.03891.015

**Figure 5—figure supplement 4.**
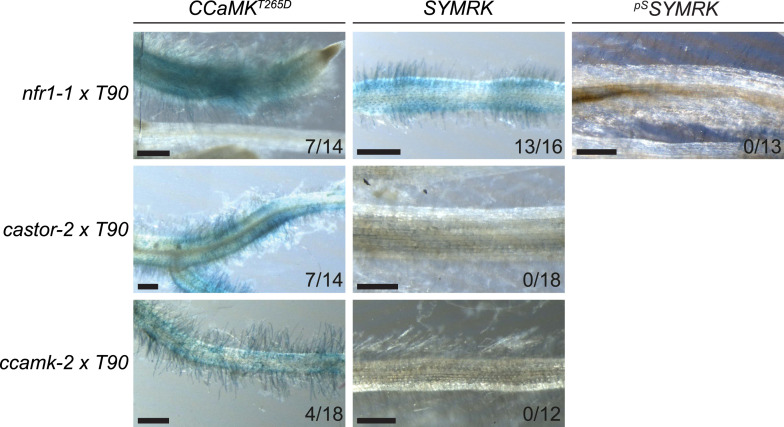
*SYMRK*-mediated activation of the symbiosis-specific T90 reporter in symbiosis-defective mutants. Hairy roots of three stable transgenic *L. japonicus* Gifu reporter lines homozygous for the T90 reporter fusion and the indicated mutant alleles transformed with *pUB:CCaMK* ^*T265D*^ (*CCaMK* ^*T265D*^, a deregulated version of CCaMK), *pUB:*SYMRK-mOrange (*SYMRK*), or *pSYMRK:*SYMRK-RFP (^*pS*^ *SYMRK*) were generated and kept on agar plates for a total of 38 dpt (see ‘Materials and methods’). The vast majority of transgenic root systems did not develop spontaneous nodules at this time point under these growth conditions. GUS activity was analysed by histochemical staining with X-Gluc at 38 dpt. Representative root sections are shown. Number of plants with detectable GUS activity per total plants is indicated. Bars, 500 μm. **DOI:** http://dx.doi.org/10.7554/eLife.03891.016

**Figure 5—figure supplement 5.**
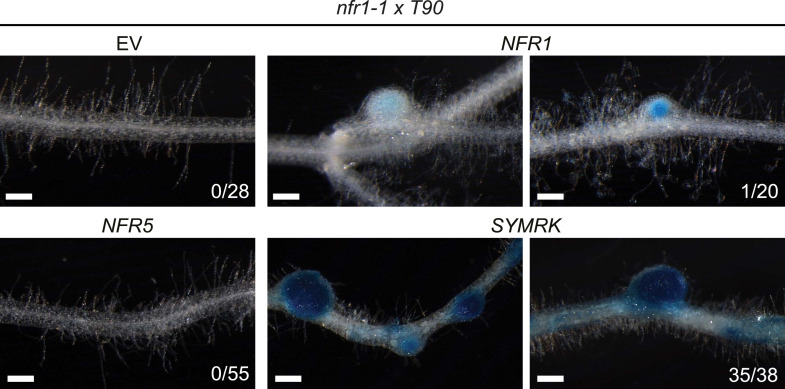
*NFR*-mediated activation of the symbiosis-specific T90 reporter in the *nfr1-1* mutant background. Hairy roots a stable transgenic *L. japonicus* Gifu reporter line homozygous for the T90 reporter fusion and the *nfr1-1* mutant allele transformed with the empty vector (EV), *pUB:NFR1-mOrange* (*NFR1*), *pUB:NFR5-mOrange* (*NFR5*), or *pUB:SYMRK-mOrange* (*SYMRK*) were generated. GUS activity was analysed by histochemical staining with X-Gluc at 60 dpt. Representative root sections are shown. Number of plants with detectable GUS activity per total number of plants is indicated. Bars, 500 μm. **DOI:** http://dx.doi.org/10.7554/eLife.03891.017

### Epistatic relationships between symbiotic *RLK* genes

We used the dominant active alleles to determine the hierarchy of the symbiotic *RLK* genes in the spontaneous nodulation and T90 activation pathways. Control roots of mutant lines transformed with the empty vector (218 root systems) or *SYMRK* driven by its own promoter (33 root systems) did not carry spontaneous nodules or nodule primordia (Figure 5; Figure 5—figure supplement 1–3). Expression of *pUB:SYMRK-mOrange* spontaneously activated the nodulation program in *nfr1-1*, *nfr5-2,* and *symrk-3* mutant roots (Figure 5; Figure 5—figure supplement 1). Spontaneous nodules on *nfr1-1* or *nfr5-2* roots overexpressing *SYMRK-mOrange* indicate that the simultaneous presence of both *NFRs* is not necessary for spontaneous *SYMRK*-mediated nodulation (Figure 5; Figure 5—figure supplement 1). Consistent with this result, overexpression of *SYMRK-mOrange* resulted in spontaneous GUS expression in the *nfr1-1* x T90 line (Gossmann et al., 2012) (Figure 5—figure supplement 4). *NFR*-mediated formation of spontaneous nodules could only be observed in the wild-type or the respective *nfr* mutant (Figure 5; Figure 5—figure supplement 2 and 3). Neither *NFR* construct spontaneously induced nodule organogenesis in a *symrk-3* (null mutant) or *symrk-10* (kinase dead mutant) background, indicating that the formation of nodules is depended on the presence of kinase-active SYMRK (Figure 5; Figure 5—figure supplement 2 and 3). Spontaneous *NFR5*-mediated nodulation was completely abolished in the *nfr1-1* mutant, demonstrating that *NFR1* is essential for this *NFR5* function (Figure 5; Figure 5—figure supplement 2). This dependence of *NFR5* on *NFR1* is further supported by the observation that overexpression of *NFR1-mOrange* and *SYMRK-mOrange* but not of *NFR5-mOrange* activated the T90 reporter in the *nfr1-1* mutant background (Figure 5—figure supplement 5). These results position *SYMRK* downstream of or at the same hierarchical level as *NFRs*. Moreover, while *SYMRK*-mediated spontaneous signalling does not require the simultaneous presence of *NFR1* and *NFR5*, *NFR5*-mediated spontaneous signalling is dependent on the presence of *NFR1*.

## Discussion

### Spontaneous signalling induced by receptor overexpression

A hallmark of the nitrogen-fixing symbiosis of legumes is the accommodation of rhizobia inside plant root cells in specialised organs—the nodules—that provide a favourable environment for nitrogen fixation. Given that the common symbiosis pathway is operating in AM symbiosis in most land plants, the discovery that expression of either of the three symbiotic *RLK* constructs from the strong *Ubiquitin* promoter leads to the spontaneous formation of nodules in transgenic *L. japonicus* roots (Figure 1, Figure 1—figure supplement 2; Figure 1—figure supplement 4; Figure 2; Figure 5, Figure 5—figure supplement 1–5) could pave the way towards the synthetic transfer of nitrogen-fixing root nodules to important non-leguminous crop species. As the symbiotic RLKs act at the entry level of root nodule symbiosis signalling, auto-active versions provide a valuable tool to study the entire nodulation pathway uncoupled from bacterial infection. Furthermore, dominant active *RLK* versions could be useful for probing and dissecting the symbiotic signalling pathway, also in those plant lineages that are presently unable to develop nitrogen fixing root nodule symbiosis.

### *SYMRK* has an active and direct role in symbiosis signalling

It has been observed that cytoplasmic streaming in root hairs of a *symrk-3* mutant did not resume after mechanical stimulation, which raised the possibility that the absence of calcium-spiking upon injection of calcium-sensitive dyes into mutant root hair cells was a pleiotropic effect of this increased touch sensitivity (Esseling et al., 2004; Miwa et al., 2006). If touch desensitisation was the only function of *SYMRK*, its overexpression would not lead to spontaneous nodule formation. We therefore unambiguously demonstrated a direct role of *SYMRK* in symbiosis signalling, while eliminating the possibility that the symbiosis defects of *symrk* mutants are due to pleiotropic effects only.

### *SYMRK* is positioned upstream of genes involved in calcium-spiking

Mutants defective for either of the common symbiosis genes *SYMRK, CASTOR, POLLUX, NENA, NUP85,* or *NUP133* produce very similar phenotypes in symbiosis, in that they abort infection at the epidermis and are impaired in calcium-spiking (Kistner et al., 2005; Miwa et al., 2006; Groth et al., 2010), which placed them at the same hierarchical level. Consequently, a genetic resolution of the relative position of the common symbiosis genes upstream of calcium-spiking was missing. Epistasis tests revealed that *SYMRK* initiates signalling upstream of other common symbiosis genes implicated in the generation and interpretation of nuclear calcium signatures (Figure 5, Figure 5—figure supplement 1 and 4). These findings support the conceptual framework in which SYMRK activates the calcium-spiking machinery and consequently the CCaMK/CYCLOPS complex, a central regulator of symbiosis-related gene expression and nodule organogenesis (Gleason et al., 2006; Tirichine et al., 2006; Singh and Parniske, 2012; Singh et al., 2014). This is in line with the observation that dominant active variants of CCaMK were able to restore nodulation and infection in *symrk* mutant backgrounds, indicating that a main function of SYMRK in symbiosis is the activation of CCaMK (Hayashi et al., 2010; Madsen et al., 2010).

### Interaction between SYMRK and the NFRs

We observed association between SYMRK and either NFR1 or NFR5 upon *NFR* overexpression in *L. japonicus* roots (Figure 4). Interestingly, under these conditions, the SYMRK-NFR association was detected in the absence of nodulation factor (Figure 4). In mammalian receptor tyrosine kinases as well as plant RLKs, ligand-induced receptor dimerization is the single most critical step in signal initiation (Li et al., 2002; Nam and Li, 2002; Schlessinger, 2002; Chinchilla et al., 2007; Schulze et al., 2010; Liu et al., 2012; Sun et al., 2013a, 2013b). However, ligand-independent dimerization of receptor tyrosine kinases mediated by specific mutations in the kinase domain (Shan et al., 2012) or by overabundance of receptor tyrosine kinases (Wei et al., 2005) results in signalling activation and is a scenario well described in the context of cancer formation (Schlessinger, 2002). Similarly, overexpression of symbiotic *RLKs* might trigger ligand-independent receptor complex formation and activation of downstream signalling, thus providing an explanation why the interaction was also detected in the absence of external symbiotic stimulation. Unfortunately, we could not address the question whether SYMRK-NFR interaction is ligand-induced at endogenous levels of *NFR* expression since NFR1 and NFR5 were difficult to detect under these conditions.

### The relationship between NFR1, NFR5 and SYMRK

We observed that *NFR5* requires *NFR1* as well as *SYMRK* for the spontaneous initiation of symbiosis signalling. This provides support for a model first put forward by Radutoiu et al. (2003), in which NFR1 and NFR5 engage in a nodulation factor perception complex. This model has received additional support through their synergistic effect on promoting cell death in *N. benthamiana* (Madsen et al., 2011; Pietraszewska-Bogiel et al., 2013). The finding that NFR1 and NFR5 interact with SYMRK upon overexpression suggests that the three RLKs engage in a receptor complex (Antolín-Llovera et al., 2014; Figure 4, Figure 4—figure supplement 1), and that this interaction might activate SYMRK for signal transduction. The observation that *SYMRK* operates independently of *NFR1* or *NFR5* brings about a new twist into current models of the signalling pathway (Downie, 2014) (Figure 5; Figure 5—figure supplement 1 and 4). *NFR1* and *NFR5* are only essential in the epidermis (Madsen et al., 2010; Hayashi et al., 2014), and it is likely that—at least partially—other members of the *LysM-RLK* gene family of *L. japonicus* (Lohmann et al., 2010) take over their role in the root cortex. *NFR1* or *NFR5* dispensability may be explained by other LysM-RLKs that might engage in alternative receptor complexes with SYMRK. Alternatively, spontaneous SYMRK-mediated signalling might be independent of any LysM-RLK, however, given the large number of LysM-RLKs in legumes (17 in *L. japonicus*; Lohmann et al., 2010), it is difficult to test the latter hypothesis conclusively.

SYMRK undergoes cleavage of its ectodomain, resulting in a truncated RLK molecule called SYMRK-ΔMLD (Antolín-Llovera et al., 2014). In competition experiments in *N. benthamiana* leaves, NFR5 binds preferentially to SYMRK-ΔMLD, which experiences rapid turnover in *N. benthamiana* and in *L. japonicus* (Antolín-Llovera et al., 2014). As our SYMRK antibody does not recognise endogenous SYMRK-ΔMLD, we were not able to assess whether overexpressed NFR1 or NFR5 also associates with this truncated SYMRK variant in *L. japonicus* roots. In a hypothetical scenario, the SYMRK-ΔMLD complex with NFR5 forms constitutively to prevent inappropriate signalling, for example in the absence of rhizobia. The recruitment of NFR1, a hypothetical signal initiation event, would be promoted by the presence of nodulation factor. Our observation that upon overexpression in *L. japonicus* both NFR1 and NFR5 seem to interact with full-length SYMRK (Figure 4) suggests the formation of a ternary complex. This hypothetical complex has dual functionality: it signals through SYMRK on one hand to activate CCaMK and through the NFR1-NFR5 complex on the other hand to trigger the infection-related parallel pathways discovered by Madsen et al. (2010) and Hayashi et al. (2010). It is possible that SYMRK has a dual—positive and negative—regulatory role: on the one hand SYMRK promotes signalling but on the other hand SYMRK-ΔMLD may be involved in preventing inappropriate signalling. A negative regulatory role would explain the exaggerated root hair response of *symrk* mutants to rhizobia (Stracke et al., 2002), since NFR1–NFR5 interaction is no longer under governance by SYMRK-ΔMLD. It has been demonstrated recently that expression of the intracellular kinase domain of *SYMRK* (*SYMRK-KD*) from *Medicago truncatula* or *Arachis hypogaea* in *M. truncatula* roots from the CaMV 35S promoter induces nodule organogenesis in the absence of rhizobia (Saha et al., 2014). However, in the presence of *Sinorhizobium meliloti*, nodules on plants overexpressing *AhSYMRK-KD* were poorly colonized and bacteria were rarely released from infection threads (Saha et al., 2014).

### Heterocomplexes between SYMRK and alternative LysM-RLKs may govern nodulation- vs mycorrhiza signalling

The origin of AM dates back to the earliest land plants (∼400 mya) and recent angiosperms maintained a conserved genetic program for the intracellular accommodation of AM fungi (Gutjahr and Parniske, 2013). During the evolution of the nitrogen-fixing root nodule symbiosis, this ancient genetic programme has been co-opted, as evidenced by the common symbiosis genes (Kistner et al., 2005). The discovery that the ancient SYMRK might act as a docking site for the recently evolved nodulation factor perception system (Antolín-Llovera et al., 2014; Figure 4, Figure 4—figure supplement 1), highlights the role of this putative interface during the recruitment of the ancestral AM signalling pathway for root nodule symbiosis. Since a LysM-RLK closely related to NFR5 has been implicated in AM signalling (Op Den Camp et al., 2011), this finding also provides a conceptual mechanism for the integration of signals from the rhizobial and fungal microsymbiont through alternative complex formation between SYMRK and NFRs or AM factor receptors.

### Specificity originates from the receptors

One question that has puzzled the community since the postulate of a common symbiosis pathway is how the decision between the developmental pathways of AM or root nodule symbiosis is made when the signalling employs identical signalling components. Models proposed involved different calcium-spiking signatures with symbiosis-specific information content (Kosuta et al., 2008) or additional yet unidentified pathways that operate in parallel to the common symbiosis pathway to mediate exclusive and appropriate signalling (Takeda et al., 2011). Our observation of differential gene activation triggered by *NFR*s and *SYMRK* provides evidence that an important decision point is directly at the level of the receptors (Figure 2 and 3). Moreover, the observation that the dominant active *SYMRK* allele activates both pathways, which is not detected by stimulation with AM fungi or rhizobia, implies the existence of negative regulatory mechanisms that prevent the activation of the inappropriate pathway upon contact with either bacterial or fungal microsymbiont. The *SYMRK*-mediated loss of signalling specificity may be explained by simultaneous complex formation of SYMRK with NFR1 and NFR5, and related LysM-RLKs that mediate recognition of signals from the AM fungus (Maillet et al., 2011; Op Den Camp et al., 2011), which results in the release of both negative regulatory mechanisms, or by an unbalanced stoichiometry of SYMRK and putative specific negative regulators of AM- and root nodule symbiosis signalling. Candidates for such regulators include the identified interactors of the kinase domains of NFR1, NFR5, and SYMRK (Kevei et al., 2007; Zhu et al., 2008; Lefebvre et al., 2010; Mbengue et al., 2010; Chen et al., 2012; Den Herder et al., 2012; Ke et al., 2012; Toth et al., 2012; Yuan et al., 2012). The loss of signalling specificity upon *SYMRK* overexpression is reminiscent of expression of the deregulated CCaMK_314_ deletion mutant that also induces spontaneous nodules and AM-related gene activation (Takeda et al., 2012). It is therefore possible that *SYMRK* overexpression imposes a deregulated state on CCaMK that is otherwise attainable artificially through the deletion of its regulatory domain.

## Materials and methods

### DNA constructs and primers

For a detailed description of the constructs and primers used in this study, please see Supplementary file 1.

### *Agrobacterium tumefaciens*-mediated transient transformation of *Nicotiana benthamiana* leaves

Transient transformation of *N. benthamiana* leaves was performed as described previously (Antolín-Llovera et al., 2014).

### Plant growth, hairy root transformation and inoculation

*L. japonicus* seed germination (Groth et al., 2010) and hairy root transformation (Charpentier et al., 2008) were performed as described previously. Plants with emerging hairy roots systems were transferred to Fahraeus medium (FP) plates containing 0.1 µM of the ethylene biosynthesis inhibitor L-α-(2-aminoethoxyvinyl)-glycine at 2.5 weeks after transformation. For spontaneous nodulation experiments, promoter activation assays, or qRT-PCR experiments, plants were transferred to sterile Weck jars containing 300 ml dried sand/vermiculite and 25 ml FP medium at 23 dpt. For co-enrichment experiments, plants were transferred to sterile Weck jars containing 300 ml dried sand/vermiculite at 23 dpt, mock treated with 20 ml FP medium or inoculated with 20 ml of a *M. loti* MAFF303099 *Ds*RED suspension in FP medium set to an OD_600_ of 0.05, and incubated for 10 days. Plants for the SYMRK- and CCaMK^T265D^-mediated T90 activation in the *nfr1-1*, *cyclops-2,* and *ccamk-2* mutants were transferred to FP plates containing 0.1 µM of the ethylene biosynthesis inhibitor L-α-(2-aminoethoxyvinyl)-glycine at 21 dpt and kept on FP plates for 17 days. Transformants of the *pSbtM1:GUS* line were directly transferred to Weck jars containing 300 ml dried sand/vermiculite and approximately 25 ml ddH_2_O at 2.5 weeks after transformation. It is important to avoid free water at the bottom of the Weck jar. Plants were grown in Weck jars in a growth chamber (16 hr light/8 hr dark; 24°C) for 1.5–6 weeks. For complementation experiments, plants were transferred from FP plates to open pots containing 300 ml dried sand/vermiculite and 75 ml FP medium at 23 dpt. After 1 week, plants were inoculated with 25 ml per pot of a *M. loti* MAFF303099 *Ds*RED suspension in FP medium set to an OD_600_ of 0.05. Roots were phenotyped 15 days after inoculation.

### Non-denaturing protein extraction from *Nicotiana benthamiana* leaves and immunoprecipitation experiments

Protein extraction and immunoprecipitation was performed as described previously (Antolín-Llovera et al., 2014).

### Non-denaturing protein extraction from *Lotus japonicus* hairy roots and immuno-enrichment experiments

Plant tissue was ground to a fine powder in liquid nitrogen with mortar and pestle. Proteins were extracted by adding 200 µl extraction buffer per 100 mg root tissue (50 mM Hepes, pH 7.5, 10 mM EDTA, 150 mM NaCl, 10% sucrose, 2 mM DTT, 0.5 mg/ml Pefabloc, 1% Triton-X 100, PhosSTOP [Roche, Germany], Plant Protease Inhibitor [P9599; Sigma–Aldrich, Germany], 1% polyvinylpolypyrrolidone). Samples were incubated for 10 min at 4°C with 20 rpm end-over mixing, and subsequently centrifuged for 15 min at 4°C and 16,000 RCF. 30 µl of each protein extract was mixed with 10 µl 4× SDS-PAGE sample buffer (input; 25% (vol/vol) 0.5 M Tris–HCl (pH 6.8), 35% (vol/vol) 20% SDS, 40% (vol/vol) 100% Glycerol, 0.03 g/ml DTT, dash of Bromphenol blue). For immuno-enrichment procedures, 30 µl RFP binder coupled to magnetic particles (rtm-20; Chromotek, Germany) were washed in wash buffer (WB; 50 mM Hepes, pH 7.5, 10 mM EDTA, 150 mM NaCl, 1% Triton-X 100). Between 500 and 1000 µl of the protein extract was added to the beads and immuno-enrichment was performed for 4 hr at 4°C with 20 rpm end-over mixing, followed by 15 min magnetic separation at 4°C. Supernatant was removed and beads were washed twice with WB. 40 µl 2× SDS-PAGE sample buffer was added to the beads and both beads and input were incubated 10 min at 56°C. After heating, beads were magnetically collected at the tube wall for 5 min and 40 µl of the supernatant (eluate) was taken. For SDS-PAGE, 20 µl of the input or eluate were loaded on each gel.

### Western blot analysis

Western blot analysis was performed as described previously (Antolín-Llovera et al., 2014).

### T90, *NIN, SbtM1,* and *SbtS* promoter analysis in *Lotus japonicus*

GUS activity originating from the activation of promoter:*GUS* reporters was visualized by X-Gluc staining as described previously (Groth et al., 2010).

### Expression analysis

Transgenic root systems of *L. japonicus* plants were harvested 40 dpt. 80 mg root fresh weight per sample was applied for total RNA extraction using the Spectrum Plant Total RNA kit (Sigma–Aldrich, Germany). For removal of genomic DNA, RNA was treated with DNase I (amplification grade DNase I, Invitrogen, Germany). RNA integrity was verified on an agarose gel and the absence of genomic DNA was confirmed by PCR. First strand cDNA synthesis was performed in 20 µl reactions with 600 ng total RNA using the SuperScript III First-Strand Synthesis SuperMix (Invitrogen, Germany) with oligo(dT) primers. qRT-PCR was performed in 20 µl reactions containing 1× SYBR Green I (Invitrogen, Germany) in a CFX96 Real-time PCR detection system (Bio-Rad, Germany). PCR program: 95°C for 2 min, 45 × (95°C for 30 s; 60°C for 30 s; 72°C for 20 s; plate read), 95°C for 10 s, melt curve 60°C–95°C: increment 0.5°C per 5 s. Expression was normalized to the reference genes *EF-1alpha* and *Ubiquitin*, and *EF-1alpha* was used as a reference to calculate the relative expression of the target genes. The empty vector samples were used as negative control. Three biological replicates were analysed in technical duplicates per treatment. A primer list can be found in the supplementary files (Supplementary file 1B).

### Statistics and data visualisation

All statistical analyses and data plots have been performed and generated with R version 3.0.2 (2013-09-25) ‘Frisbee Sailing’ (R Development Core Team, 2008) and the packages ‘Hmisc’ (Harrell, 2014), ‘agricolae’ (Mendiburu de, 2014), ‘car’ (Fox and Weisberg, 2011), ‘multcompView’ (Graves et al., 2012) and ‘multcomp’ (Hothorn et al., 2008). For statistical analysis of the numbers of nodules, nodule primordia, or total organogenesis events, a Kruskal–Wallis test was applied followed by false discovery rate correction. Quantitative real-time PCR data were power transformed with the Box–Cox transformation and a one-way ANOVA followed by a Dunnett's test was performed, in which every treatment was compared to the empty vector samples.

## Additional files

Supplementary file 1. (**A**) Constructs. (**B**) Oligonucleotides.
**DOI:** http://dx.doi.org/10.7554/eLife.03891.018

## References

[bib1] Antolín-Llovera M, Ried MK, Parniske M (2014). Cleavage of the SYMBIOSIS RECEPTOR-LIKE KINASE ectodomain promotes complex formation with Nod Factor Receptor 5. Current Biology.

[bib2] Broghammer A, Krusell L, Blaise M, Sauer J, Sullivan JT, Maolanon N, Vinther M, Lorentzen A, Madsen EB, Jensen KJ, Roepstorff P, Thirup S, Ronson CW, Thygesen MB, Stougaard J (2012). Legume receptors perceive the rhizobial lipochitin oligosaccharide signal molecules by direct binding. Proceedings of the National Academy of Sciences of USA.

[bib3] Charpentier M, Bredemeier R, Wanner G, Takeda N, Schleiff E, Parniske M (2008). *Lotus japonicus* CASTOR and POLLUX are ion channels essential for perinuclear calcium spiking in legume root endosymbiosis. The Plant Cell.

[bib4] Chen T, Zhu H, Ke D, Cai K, Wang C, Gou H, Hong Z, Zhang Z (2012). A MAP kinase kinase interacts with SymRK and regulates nodule organogenesis in *Lotus japonicus*. Plant Cell.

[bib78] Chinchilla D, Zipfel C, Robatzek S, Kemmerling B, Nürnberger T, Jones JD, Felix G, Boller T (2007). A flagellin-induced complex of the receptor FLS2 and BAK1 initiates plant defence. Nature.

[bib5] Den Herder G, Yoshida S, Antolín-Llovera M, Ried MK, Parniske M (2012). *Lotus japonicus* E3 ligase SEVEN IN ABSENTIA4 destabilizes the symbiosis receptor-like kinase SYMRK and negatively regulates rhizobial infection. The Plant Cell.

[bib6] Downie JA (2014). Legume nodulation. Current Biology.

[bib7] Ehrhardt DW, Wais R, Long SR (1996). Calcium spiking in plant root hairs responding to *Rhizobium* nodulation signals. Cell.

[bib8] Esseling JJ, Lhuissier FG, Emons AM (2004). A nonsymbiotic root hair tip growth phenotype in *NORK*-mutated legumes: implications for nodulation factor-induced signaling and formation of a multifaceted root hair pocket for bacteria. The Plant Cell.

[bib9] Fox J, Weisberg S (2011). An {R} companion to applied regression.

[bib11] Genre A, Chabaud M, Balzergue C, Puech-Pages V, Novero M, Rey T, Fournier J, Rochange S, Becard G, Bonfante P, Barker DG (2013). Short-chain chitin oligomers from arbuscular mycorrhizal fungi trigger nuclear Ca^2+^ spiking in *Medicago truncatula* roots and their production is enhanced by strigolactone. The New Phytologist.

[bib10] Genre A, Chabaud M, Timmers T, Bonfante P, Barker DG (2005). Arbuscular mycorrhizal fungi elicit a novel intracellular apparatus in *Medicago truncatula* root epidermal cells before infection. The Plant Cell.

[bib12] Gleason C, Chaudhuri S, Yang T, Munoz A, Poovaiah BW, Oldroyd GE (2006). Nodulation independent of rhizobia induced by a calcium-activated kinase lacking autoinhibition. Nature.

[bib13] Gossmann JA, Markmann K, Brachmann A, Rose LE, Parniske M (2012). Polymorphic infection and organogenesis patterns induced by a *Rhizobium leguminosarum* isolate from *Lotus* root nodules are determined by the host genotype. The New Phytologist.

[bib14] Graves S, Piepho HP, Selzer L (2012). multcompView: Visualizations of paired comparisons. R package version 0.1-5.

[bib15] Groth M, Takeda N, Perry J, Uchida H, Draxl S, Brachmann A, Sato S, Tabata S, Kawaguchi M, Wang TL, Parniske M (2010). *NENA*, a *Lotus japonicus* homolog of *Sec13*, is required for rhizodermal infection by arbuscular mycorrhiza fungi and rhizobia but dispensable for cortical endosymbiotic development. The Plant Cell.

[bib16] Gutjahr C, Parniske M (2013). Cell and developmental biology of arbuscular mycorrhiza symbiosis. Annual Review of Cell and Developmental Biology.

[bib17] Harrell FE (2014). Hmisc: Harrell Miscellaneous. R package version 3.14-0.

[bib18] Hayashi T, Banba M, Shimoda Y, Kouchi H, Hayashi M, Imaizumi-Anraku H (2010). A dominant function of CCaMK in intracellular accommodation of bacterial and fungal endosymbionts. The Plant Journal.

[bib19] Hayashi T, Shimoda Y, Sato S, Tabata S, Imaizumi-Anraku H, Hayashi M (2014). Rhizobial infection does not require cortical expression of upstream common symbiosis genes responsible for the induction of Ca^2+^ spiking. The Plant Journal.

[bib20] Hohnjec N, Vieweg MF, Puhler A, Becker A, Kuster H (2005). Overlaps in the transcriptional profiles of *Medicago truncatula* roots inoculated with two different *Glomus* fungi provide insights into the genetic program activated during arbuscular mycorrhiza. Plant Physiology.

[bib21] Horvath B, Yeun LH, Domonkos A, Halasz G, Gobbato E, Ayaydin F, Miro K, Hirsch S, Sun J, Tadege M, Ratet P, Mysore KS, Ane JM, Oldroyd GE, Kalo P (2011). *Medicago truncatula IPD3* is a member of the common symbiotic signaling pathway required for rhizobial and mycorrhizal symbioses. Molecular Plant-microbe Interactions.

[bib79] Hothorn T, Bretz F, Westfall P (2008). Simultaneous inference in general parametric models. Biometrical Journal.

[bib22] Imaizumi-Anraku H, Takeda N, Charpentier M, Perry J, Miwa H, Umehara Y, Kouchi H, Murakami Y, Mulder L, Vickers K, Pike J, Downie JA, Wang T, Sato S, Asamizu E, Tabata S, Yoshikawa M, Murooka Y, Wu GJ, Kawaguchi M, Kawasaki S, Parniske M, Hayashi M (2005). Plastid proteins crucial for symbiotic fungal and bacterial entry into plant roots. Nature.

[bib23] Kanamori N, Madsen LH, Radutoiu S, Frantescu M, Quistgaard EM, Miwa H, Downie JA, James EK, Felle HH, Haaning LL, Jensen TH, Sato S, Nakamura Y, Tabata S, Sandal N, Stougaard J (2006). A nucleoporin is required for induction of Ca^2+^ spiking in legume nodule development and essential for rhizobial and fungal symbiosis. Proceedings of the National Academy of Sciences of USA.

[bib24] Ke D, Fang Q, Chen C, Zhu H, Chen T, Chang X, Yuan S, Kang H, Ma L, Hong Z, Zhang Z (2012). The small GTPase ROP6 interacts with NFR5 and is involved in nodule formation in *Lotus japonicus*. Plant Physiology.

[bib25] Kevei Z, Lougnon G, Mergaert P, Horvath GV, Kereszt A, Jayaraman D, Zaman N, Marcel F, Regulski K, Kiss GB, Kondorosi A, Endre G, Kondorosi E, Ane JM (2007). 3-hydroxy-3-methylglutaryl coenzyme a reductase 1 interacts with NORK and is crucial for nodulation in Medicago truncatula. The Plant Cell.

[bib26] Kistner C, Winzer T, Pitzschke A, Mulder L, Sato S, Kaneko T, Tabata S, Sandal N, Stougaard J, Webb KJ, Szczyglowski K, Parniske M (2005). Seven *Lotus japonicus* genes required for transcriptional reprogramming of the root during fungal and bacterial symbiosis. The Plant Cell.

[bib27] Kosuta S, Hazledine S, Sun J, Miwa H, Morris RJ, Downie JA, Oldroyd GE (2008). Differential and chaotic calcium signatures in the symbiosis signaling pathway of legumes. Proceedings of the National Academy of Sciences of USA.

[bib28] Kosuta S, Held M, Hossain MS, Morieri G, Macgillivary A, Johansen C, Antolin-Llovera M, Parniske M, Oldroyd GE, Downie AJ, Karas B, Szczyglowski K (2011). *Lotus japonicus symRK-14* uncouples the cortical and epidermal symbiotic program. The Plant Journal.

[bib29] Lefebvre B, Timmers T, Mbengue M, Moreau S, Herve C, Toth K, Bittencourt-Silvestre J, Klaus D, Deslandes L, Godiard L, Murray JD, Udvardi MK, Raffaele S, Mongrand S, Cullimore J, Gamas P, Niebel A, Ott T (2010). A remorin protein interacts with symbiotic receptors and regulates bacterial infection. Proceedings of the National Academy of Sciences of USA.

[bib30] Li J, Chory J (1997). A putative leucine-rich repeat receptor kinase involved in brassinosteroid signal transduction. Cell.

[bib31] Li J, Wen J, Lease KA, Doke JT, Tax FE, Walker JC (2002). BAK1, an *Arabidopsis* LRR receptor-like protein kinase, interacts with BRI1 and modulates brassinosteroid signaling. Cell.

[bib32] Liu J, Blaylock LA, Endre G, Cho J, Town CD, Vandenbosch KA, Harrison MJ (2003). Transcript profiling coupled with spatial expression analyses reveals genes involved in distinct developmental stages of an arbuscular mycorrhizal symbiosis. The Plant Cell.

[bib33] Liu T, Liu Z, Song C, Hu Y, Han Z, She J, Fan F, Wang J, Jin C, Chang J, Zhou JM, Chai J (2012). Chitin-induced dimerization activates a plant immune receptor. Science.

[bib34] Lohmann GV, Shimoda Y, Nielsen MW, Jorgensen FG, Grossmann C, Sandal N, Sorensen K, Thirup S, Madsen LH, Tabata S, Sato S, Stougaard J, Radutoiu S (2010). Evolution and regulation of the *Lotus japonicus LysM receptor* gene family. Molecular Plant-microbe Interactions.

[bib37] Madsen EB, Antolín-Llovera M, Grossmann C, Ye J, Vieweg S, Broghammer A, Krusell L, Radutoiu S, Jensen ON, Stougaard J, Parniske M (2011). Autophosphorylation is essential for the in vivo function of the *Lotus japonicus* Nod factor receptor 1 and receptor-mediated signalling in cooperation with Nod factor receptor 5. The Plant Journal.

[bib35] Madsen EB, Madsen LH, Radutoiu S, Olbryt M, Rakwalska M, Szczyglowski K, Sato S, Kaneko T, Tabata S, Sandal N, Stougaard J (2003). A receptor kinase gene of the LysM type is involved in legume perception of rhizobial signals. Nature.

[bib36] Madsen LH, Tirichine L, Jurkiewicz A, Sullivan JT, Heckmann AB, Bek AS, Ronson CW, James EK, Stougaard J (2010). The molecular network governing nodule organogenesis and infection in the model legume *Lotus japonicus*. Nature Communications.

[bib38] Maekawa T, Kusakabe M, Shimoda Y, Sato S, Tabata S, Murooka Y, Hayashi M (2008). Polyubiquitin promoter-based binary vectors for overexpression and gene silencing in *Lotus japonicus*. Molecular Plant-Microbe Interactions.

[bib39] Maillet F, Poinsot V, Andre O, Puech-Pages V, Haouy A, Gueunier M, Cromer L, Giraudet D, Formey D, Niebel A, Martinez EA, Driguez H, Becard G, Denarie J (2011). Fungal lipochitooligosaccharide symbiotic signals in arbuscular mycorrhiza. Nature.

[bib40] Markmann K, Giczey G, Parniske M (2008). Functional adaptation of a plant receptor-kinase paved the way for the evolution of intracellular root symbioses with bacteria. Plos Biology.

[bib41] Mbengue M, Camut S, De Carvalho-Niebel F, Deslandes L, Froidure S, Klaus-Heisen D, Moreau S, Rivas S, Timmers T, Herve C, Cullimore J, Lefebvre B (2010). The *Medicago truncatula* E3 ubiquitin ligase PUB1 interacts with the LYK3 symbiotic receptor and negatively regulates infection and nodulation. The Plant Cell.

[bib42] Mendiburu de F (2014). agricolae: statistical procedures for agricultural research. R package version 1.1-7.

[bib43] Miller JB, Pratap A, Miyahara A, Zhou L, Bornemann S, Morris RJ, Oldroyd GE (2013). Calcium/Calmodulin-dependent protein kinase is negatively and positively regulated by calcium, providing a mechanism for decoding calcium responses during symbiosis signaling. The Plant Cell.

[bib44] Miwa H, Sun J, Oldroyd GE, Downie JA (2006). Analysis of Nod-factor-induced calcium signaling in root hairs of symbiotically defective mutants of *Lotus japonicus*. Molecular Plant-Microbe Interactions.

[bib45] Nam KH, Li J (2002). BRI1/BAK1, a receptor kinase pair mediating brassinosteroid signaling. Cell.

[bib46] Oldroyd GE (2013). Speak, friend, and enter: signalling systems that promote beneficial symbiotic associations in plants. Nature Reviews Microbiology.

[bib47] Op Den Camp R, Streng A, De Mita S, Cao Q, Polone E, Liu W, Ammiraju JS, Kudrna D, Wing R, Untergasser A, Bisseling T, Geurts R (2011). LysM-type mycorrhizal receptor recruited for rhizobium symbiosis in nonlegume *Parasponia*. Science.

[bib48] Ovchinnikova E, Journet EP, Chabaud M, Cosson V, Ratet P, Duc G, Fedorova E, Liu W, Den Camp RO, Zhukov V, Tikhonovich I, Borisov A, Bisseling T, Limpens E (2011). IPD3 controls the formation of nitrogen-fixing symbiosomes in pea and *Medicago* Spp. Molecular Plant-Microbe Interactions.

[bib49] Pietraszewska-Bogiel A, Lefebvre B, Koini MA, Klaus-Heisen D, Takken FL, Geurts R, Cullimore JV, Gadella TW (2013). Interaction of *Medicago truncatula* lysin motif receptor-like kinases, NFP and LYK3, produced in *Nicotiana benthamiana* induces defence-like responses. PLOS ONE.

[bib50] R Development Core Team (2008). R: a language and environment for statistical computing. R Foundation for Statistical Computing.

[bib51] Radutoiu S, Madsen LH, Madsen EB, Felle HH, Umehara Y, Gronlund M, Sato S, Nakamura Y, Tabata S, Sandal N, Stougaard J (2003). Plant recognition of symbiotic bacteria requires two LysM receptor-like kinases. Nature.

[bib52] Radutoiu S, Madsen LH, Madsen EB, Jurkiewicz A, Fukai E, Quistgaard EM, Albrektsen AS, James EK, Thirup S, Stougaard J (2007). LysM domains mediate lipochitin-oligosaccharide recognition and *Nfr* genes extend the symbiotic host range. The EMBO Journal.

[bib53] Saha S, Dutta A, Bhattacharya A, Dasgupta M (2014). Intracellular catalytic domain of Symbiosis Receptor Kinase (SYMRK) hyperactivates spontaneous nodulation in absence of rhizobia. Plant Physiology.

[bib54] Saito K, Yoshikawa M, Yano K, Miwa H, Uchida H, Asamizu E, Sato S, Tabata S, Imaizumi-Anraku H, Umehara Y, Kouchi H, Murooka Y, Szczyglowski K, Downie JA, Parniske M, Hayashi M, Kawaguchi M (2007). NUCLEOPORIN85 is required for calcium spiking, fungal and bacterial symbioses, and seed production in *Lotus japonicus*. The Plant Cell.

[bib55] Schauser L, Roussis A, Stiller J, Stougaard J (1999). A plant regulator controlling development of symbiotic root nodules. Nature.

[bib56] Schlessinger J (2002). Ligand-induced, receptor-mediated dimerization and activation of EGF receptor. Cell.

[bib57] Schulze B, Mentzel T, Jehle AK, Mueller K, Beeler S, Boller T, Felix G, Chinchilla D (2010). Rapid heteromerization and phosphorylation of ligand-activated plant transmembrane receptors and their associated kinase BAK1. The Journal of Biological Chemistry.

[bib58] Shan Y, Eastwood MP, Zhang X, Kim ET, Arkhipov A, Dror RO, Jumper J, Kuriyan J, Shaw DE (2012). Oncogenic mutations counteract intrinsic disorder in the EGFR kinase and promote receptor dimerization. Cell.

[bib60] Singh S, Katzer K, Lambert J, Cerri M, Parniske M (2014). CYCLOPS, a DNA-binding transcriptional activator, orchestrates symbiotic root nodule development. Cell Host & Microbe.

[bib59] Singh S, Parniske M (2012). Activation of calcium- and calmodulin-dependent protein kinase (CCaMK), the central regulator of plant root endosymbiosis. Current Opinion in Plant Biology.

[bib61] Soyano T, Kouchi H, Hirota A, Hayashi M (2013). Nodule inception directly targets *NF-Y* subunit genes to regulate essential processes of root nodule development in Lotus japonicus. PLOS Genetics.

[bib62] Stracke S, Kistner C, Yoshida S, Mulder L, Sato S, Kaneko T, Tabata S, Sandal N, Stougaard J, Szczyglowski K, Parniske M (2002). A plant receptor-like kinase required for both bacterial and fungal symbiosis. Nature.

[bib64] Sun Y, Han Z, Tang J, Hu Z, Chai C, Zhou B, Chai J (2013a). Structure reveals that BAK1 as a co-receptor recognizes the BRI1-bound brassinolide. Cell Research.

[bib63] Sun Y, Li L, Macho AP, Han Z, Hu Z, Zipfel C, Zhou JM, Chai J (2013b). Structural basis for flg22-induced activation of the *Arabidopsis* FLS2-BAK1 immune complex. Science.

[bib66] Takeda N, Haage K, Sato S, Tabata S, Parniske M (2011). Activation of a *Lotus japonicus* subtilase gene during arbuscular mycorrhiza is dependent on the common symbiosis genes and two *cis*-active promoter regions. Molecular Plant-microbe Interactions.

[bib67] Takeda N, Maekawa T, Hayashi M (2012). Nuclear-localized and deregulated calcium- and calmodulin-dependent protein kinase activates rhizobial and mycorrhizal responses in *Lotus japonicus*. Plant Cell.

[bib65] Takeda N, Sato S, Asamizu E, Tabata S, Parniske M (2009). Apoplastic plant subtilases support arbuscular mycorrhiza development in *Lotus japonicus*. The Plant Journal.

[bib68] Tirichine L, Imaizumi-Anraku H, Yoshida S, Murakami Y, Madsen LH, Miwa H, Nakagawa T, Sandal N, Albrektsen AS, Kawaguchi M, Downie A, Sato S, Tabata S, Kouchi H, Parniske M, Kawasaki S, Stougaard J (2006). Deregulation of a Ca^2+^/calmodulin-dependent kinase leads to spontaneous nodule development. Nature.

[bib69] Toth K, Stratil TF, Madsen EB, Ye J, Popp C, Antolín-Llovera M, Grossmann C, Jensen ON, Schussler A, Parniske M, Ott T (2012). Functional domain analysis of the Remorin protein LjSYMREM1 in *Lotus japonicus*. PLOS ONE.

[bib70] Venkateshwaran M, Cosme A, Han L, Banba M, Satyshur KA, Schleiff E, Parniske M, Imaizumi-Anraku H, Ane JM (2012). The recent evolution of a symbiotic ion channel in the legume family altered ion conductance and improved functionality in calcium signaling. The Plant Cell.

[bib71] Webb KJ, Skot L, Nicholson MN, Jorgensen B, Mizen S (2000). *Mesorhizobium loti* increases root-specific expression of a calcium-binding protein homologue identified by promoter tagging in *Lotus japonicus*. Molecular Plant-Microbe Interactions.

[bib72] Wei X, Ni S, Correll PH (2005). Uncoupling ligand-dependent and -independent mechanisms for mitogen-activated protein kinase activation by the murine Ron receptor tyrosine kinase. The Journal of Biological Chemistry.

[bib73] Yano K, Yoshida S, Muller J, Singh S, Banba M, Vickers K, Markmann K, White C, Schuller B, Sato S, Asamizu E, Tabata S, Murooka Y, Perry J, Wang TL, Kawaguchi M, Imaizumi-Anraku H, Hayashi M, Parniske M (2008). CYCLOPS, a mediator of symbiotic intracellular accommodation. Proceedings of the National Academy of Sciences of USA.

[bib74] Yoro E, Suzaki T, Toyokura K, Miyazawa H, Fukaki H, Kawaguchi M (2014). A positive regulator of nodule organogenesis, NODULE INCEPTION, acts as a negative regulator of rhizobial infection in *Lotus japonicus*. Plant Physiology.

[bib75] Yuan S, Zhu H, Gou H, Fu W, Liu L, Chen T, Ke D, Kang H, Xie Q, Hong Z, Zhang Z (2012). A ubiquitin ligase of symbiosis receptor kinase involved in nodule organogenesis. Plant Physiology.

[bib76] Zhu H, Chen T, Zhu M, Fang Q, Kang H, Hong Z, Zhang Z (2008). A novel ARID DNA-binding protein interacts with SymRK and is expressed during early nodule development in *Lotus japonicus*. Plant Physiology.

[bib77] Zipfel C, Kunze G, Chinchilla D, Caniard A, Jones JD, Boller T, Felix G (2006). Perception of the bacterial PAMP EF-Tu by the receptor EFR restricts *Agrobacterium*-mediated transformation. Cell.

