# Supplementary material for: Spontaneous symbiotic reprogramming of plant roots triggered by receptor-like kinases
Source: eLife. 2014 Nov 25;3:e03891. doi: 10.7554/eLife.03891 (PMC4243133; doi:10.7554/eLife.03891)
Supplement: Supplementary file 1. — (A) Constructs. (B) Oligonucleotides. DOI: http://dx.doi.org/10.7554/eLife.03891.018 [file elife-03891-supp1.docx]

# Supplemental File 1

## Supplemental File 1 A: Constructs.

Constructs labelled with “GG” were generated via Golden Gate cloning (Binder et al., 2014).

| Entry clones / Golden Gate Level I & Level II plasmids (LI & LII) | | |
| --- | --- | --- |
| **Name** | **Description** |  |
| *pENTR:NFR1* | Phusion PCR product amplified from *p35S:NFR1-YFPv* with caccNFR1_fwd and NFR1_rev; cloned into pENTR/D-TOPO (Invitrogen) via TOPO reaction | |
| *pENTR:NFR5* | Phusion PCR product amplified with caccNFR5_fwd and NFR5_rev; cloned into pENTR/D-TOPO (Invitrogen) via TOPO reaction | |
| *pENTR:NFR1-mOrange* | Phusion PCR product amplified from *p35S:NFR1-mOrange* with caccNFR1_fwd and mOrange_STOP; cloned into pENTR/D-TOPO (Invitrogen) via TOPO reaction | |
| *pENTR:NFR5-mOrange* | Phusion PCR product amplified from *p35S:NFR5-mOrange* with caccNFR5_fwd and mOrange_STOP; cloned into pENTR/D-TOPO (Invitrogen) via TOPO reaction | |
| *pENTR:EFR-mOrange* | Phusion PCR product amplified from *p35S:EFR-mOrange* with EFR_SP_fwd and mOrange_STOP | |
| LI C-D *SYMRK (GG)* | LI element containing *SYMRK* | |
| LII F 2-3 *pUBi:SYMRK (GG)* | Assembled by BsaI cut ligation from:  LI A-B pUBi + LI dy B-C + LI C-D *SYMRK* + LI dy D-E + LI E-F nos-T + LI dy F-G + LII F 2-3 | |
| LII F 2-3 *pUBi:SYMRK-mOrange (GG)* | Assembled by BsaI cut ligation from:  LI A-B pUBi + LI dy B-C + LI C-D *SYMRK* + LI D-E mOrange + LI E-F nos-T + LI dy F-G + LII F 2-3 | |
| Plasmids for *N. benthamiana* transformation and cloning | | |
| **Name** | **Description** | |
| *p35S:GW-mOrange* | (Bayle et al., 2008) | |
| *p35S:SYMRK-mOrange* | (Den Herder et al., 2012) | |
| *p35S:NFR1-mOrange* | LR-reaction of *pENTR:NFR1* and *p35S:GW-mOrange* | |
| *p35S:NFR1-YFPv* | (Antolín-Llovera et al., 2014) | |
| *p35S:NFR5-mOrange* | LR reaction of *pENTR:NFR5* and *p35S:GW-mOrange* | |
| *p35S:NFR5-YFPv* | (Antolín-Llovera et al., 2014) | |
| *p35S:BRI1-YFPv* | (Mbengue et al., 2010) | |
|  | | |
| Plasmids for hairy root transformation of *L. japonicus* | | |
| **Name** | **Description** | |
| *pUB:GW-GFP* | (Maekawa et al., 2008) | |
| *pUB:SYMRK-mOrange* | (Antolín-Llovera et al., 2014) | |
| *pUB:EFR-mOrange* | LR reaction of *pENTR:gEFR-mOrange* and *pUB:GW_GFP* | |
| *pUB:NFR1-mOrange* | LR reaction of *pENTR:NFR1-mOrange* (cut with ApaL1) and *pUB:GW-GFP* | |
| *pUB:NFR5-mOrange* | LR reaction of *pENTR:NFR5-mOrange* (cut with ApaL1) and *pUB:GW-GFP* | |
| *pSYMRK:SYMRK-RFP* | (Kosuta et al., 2011) | |
| *pUBi:SYMRK (GG)* | Assembled by BpiI cut ligation from:  LII dy 1-2 + LII F 2-3 *pUBi:SYMRK* + LII dy 3-4 + LII F 5-6 *p35S:GFP* + LIII β F A-B | |
| *pUBi:SYMRK-mOrange (GG)* | Assembled by BpiI cut ligation from:  LII dy 1-2 + LII F 2-3 *pUBi:SYMRK:mOrange* + LII dy 3-4 + LII F 5-6 *p35S:GFP* + LIII β F A-B | |

## Supplemental File 1B: Oligonucleotides

| Expression analysis | | | |
| --- | --- | --- | --- |
| **Target sequence** |  | | **Primer sequence** |
| *Ubiquitin*  *EF1alpha*  *NIN*  *SbtS* |  | | (Takeda et al., 2009) |
| *SbtM1*  *Bcp1* | | (Groth et al., 2013) | |
| Plasmid construction |  | |  |
| **Name** |  | | **Primer sequence** |
| caccNFR1_fwd | forward | | 5’-caccATGAAGCTAAAAACTGGTCTACTT-3’ |
| NFR1_rev | reverse | | 5’-TCTCACAGACAGTAAATTTATGA-3’ |
| caccNFR5_fwd | forward | | 5’-caccATGGCTGTCTTCTTTCTTACCTCT-3’ |
| NFR5_rev | reverse | | 5’-ACGTGCAGTAATGGAAGTCACA-3’ |
| mOrange_STOP | reverse | | 5’-TTACTTGTACAGCTCGTCCATGC-3’ |
| EFR_SP_fwd | forward | | 5’-caccATGAAGCTGTCCTTTTCACTTG-3’ |

# Supplemental Reference

Binder A, Lambert J, Morbitzer R, Popp C, Ott T, Lahaye T & Parniske M. 2014. A modular plasmid assembly kit for multigene expression, gene silencing and silencing rescue in plants. *PLoS One* **9**:e88218. doi: 10.1371/journal.pone.0088218.

Bayle V, Nussaume L & Bhat RA. 2008. Combination of novel green fluorescent protein mutant TSapphire and DsRed variant mOrange to set up a versatile in planta FRET-FLIM assay. *Plant Physiol.* **148**:51-60. doi: 10.1104/pp.108.117358.

Den Herder G, Yoshida S, Antolin-Llovera M, Ried MK & Parniske M. 2012. *Lotus japonicus* E3 ligase SEVEN IN ABSENTIA4 destabilizes the symbiosis receptor-like kinase SYMRK and negatively regulates rhizobial infection. *Plant Cell* **24**:1691-707. doi: 10.1105/tpc.110.082248.

Antolín-Llovera M, Ried MK & Parniske M. 2014. Cleavage of the SYMBIOSIS RECEPTOR-LIKE KINASE ectodomain promotes complex formation with Nod Factor Receptor 5. *Curr. Biol.* **24**:422-7. doi: 10.1016/j.cub.2013.12.053.

Mbengue M, Camut S, De Carvalho-Niebel F, Deslandes L, Froidure S, Klaus-Heisen D, Moreau S, Rivas S, Timmers T, Herve C, Cullimore J & Lefebvre B. 2010. The *Medicago truncatula* E3 ubiquitin ligase PUB1 interacts with the LYK3 symbiotic receptor and negatively regulates infection and nodulation. *Plant Cell* **22**:3474-88. doi: 10.1105/tpc.110.075861.

Maekawa T, Kusakabe M, Shimoda Y, Sato S, Tabata S, Murooka Y & Hayashi M. 2008. Polyubiquitin promoter-based binary vectors for overexpression and gene silencing in *Lotus japonicus*. *Mol. Plant Microbe Interact.* **21**:375-82. doi: 10.1094/MPMI-21-4-0375.

Kosuta S, Held M, Hossain MS, Morieri G, Macgillivary A, Johansen C, Antolin-Llovera M, Parniske M, Oldroyd GE, Downie AJ, Karas B & Szczyglowski K. 2011. *Lotus japonicus symRK-14* uncouples the cortical and epidermal symbiotic program. *Plant J.* **67**:929-40. doi: 10.1111/j.1365-313X.2011.04645.x.

Takeda N, Sato S, Asamizu E, Tabata S & Parniske M. 2009. Apoplastic plant subtilases support arbuscular mycorrhiza development in *Lotus japonicus*. *Plant J.* **58**:766-77. doi: 10.1111/j.1365-313X.2009.03824.x.

Groth M, Kosuta S, Gutjahr C, Haage K, Hardel SL, Schaub M, Brachmann A, Sato S, Tabata S, Findlay K, Wang TL & Parniske M. 2013. Two *Lotus japonicus* symbiosis mutants impaired at distinct steps of arbuscule development. *Plant J.* **75**:117-29. doi: 10.1111/tpj.12220.
